# Success of medicaments and techniques for pulpotomy of primary teeth: An overview of systematic reviews

**DOI:** 10.1111/ipd.12963

**Published:** 2022-04-26

**Authors:** Nitesh Tewari, Shubhi Goel, Vijay Prakash Mathur, Anne C. O’Connell, Riya Marie Johnson, Morankar Rahul, Farheen Sultan, Mridula Goswami, Sukeshana Srivastav, Priyanshi Ritwik

**Affiliations:** ^1^ 28730 Division of Pedodontics and Preventive Dentistry Centre for Dental Education and Research All India Institute of Medical Sciences New Delhi India; ^2^ Dublin Dental University Hospital, Trinity College (University of Dublin) Dublin Ireland; ^3^ 28730 Division of Orthodontics and Dentofacial Deformities Centre for Dental Education and Research All India Institute of Medical Sciences New Delhi India; ^4^ 162104 Pedodontics and Preventive Dentistry Maulana Azad Institute of Dental Sciences New Delhi India; ^5^ University of Texas Houston School of Dentistry Houston Texas USA

**Keywords:** Apexification, laser, primary tooth, pulpal medicaments, systematic review, therapy

## Abstract

**Background:**

Pulpotomy is an effective, vital pulp therapy procedure for caries‐affected or traumatized primary teeth. Though its efficacy is widely accepted, the superiority of medicaments and techniques remains debatable.

**Aim:**

The aims of this review were to compare the success rates of various pulpotomy medicaments or techniques, assess the methodological quality of reviews, and grade the level of evidence for each comparison.

**Design:**

This review followed the principles of evidence‐based medicine and recommendations for the overview of systematic reviews. An a priori protocol was registered in the International Prospective Register of Systematic Reviews (PROSPERO; CRD42021244489). A comprehensive literature search was performed by two reviewers, and studies were selected from various databases according to predefined criteria. Two reviewers independently used a self‐designed pilot‐tested form to extract data from the selected studies. A quality analysis was performed using A MeaSurement Tool to Assess systematic Reviews‐2 (AMSTAR‐2) and the ROBIS tool. Reporting characteristics and overlap of the primary studies were also assessed. We used modified Köhler's criteria for evaluating the quality of evidence for outcomes of included systematic reviews and meta‐analyses.

**Results:**

The scrutiny of 62 full‐text articles resulted in the inclusion of eight systematic reviews. The quality of four of the reviews was found to be critically low, and the overlap of primary studies in the meta‐analyses was found to be high. Pulpotomy medicaments/techniques, except calcium hydroxide, had success rates of more than 80% for all domains and time periods. Most of the comparisons revealed no differences in the clinical, radiographic, or overall success rates. Mineral trioxide aggregate, however, was found to have better radiographic and overall success rates than calcium hydroxide at periods greater than 12 and 18 months. It also had a greater radiographic success rate than full‐strength/1:5 diluted and full‐strength formocresol at 24 months. Formocresol was found to have better overall success rates than calcium hydroxide at all time periods and better radiographic success rates at 12 months. Only 12 of the 63 comparisons had suggestive or weak evidence, whereas all others had either negligible evidence or insufficient data.

**Conclusions:**

The pulpotomy medicaments/techniques, except calcium hydroxide, showed success rates of more than 80%, whereas most comparisons revealed no differences. Mineral trioxide aggregate, however, was found to be better than calcium hydroxide and formocresol in several respects. This study highlights the lack of evidence regarding the choice of pulpotomy agents for the treatment of caries‐affected primary teeth and elucidates the domains that require primary studies in the future.

**Why this paper is important to paediatric dentists**

- This overview of systematic reviews presents the level of evidence in the comparisons of various agents and techniques used for pulpotomy of primary teeth. This can benefit paediatric dentists by providing guidance for the selection of the most appropriate technique.
- This paper identifies the comparisons that have low‐quality evidence or require future research. This can benefit future paediatric dental researchers in generating the evidence that is currently lacking.
- The recommendations developed in this paper will help in guiding evidence‐based paediatric endodontics.

## 1 INTRODUCTION

Pulpotomy is regarded as an effective, vital pulp therapy procedure for primary teeth with pulp exposure due to dental caries or trauma. It involves the complete removal of infected or inflamed coronal pulp and preservation of the radicular pulp by a suitable technique or placement of a medicament, followed by an adequate coronal seal.^1^ Though the clinical and radiographic success rates of this procedure are well established, the comparative efficacy of the different pulpotomy techniques and medicaments remains debatable. Recent advances in the understanding of pulp biology, the regeneration of the pulp–dentin complex, and the diverse interactions between the conventional and newer biomaterials have resulted in the escalation of research related to the pulpotomy of primary teeth.^2^ Although the molecular signaling mechanisms are being deciphered, the efficacy of techniques and medicaments is being evaluated through prospective studies.

The American Academy of Pediatric Dentistry, in its guidelines for vital pulp therapy procedures, has recommended mineral trioxide aggregate (MTA) and formocresol (FC) as materials of choice for the pulpotomy of teeth expected to last for 24 months or more, with all other materials having conditional recommendations.^1^ In 2021, the International Association of Paediatric Dentistry recommended MTA, Biodentine^®^, and FC as pulpotomy medicaments, along with highlighting the carcinogenic potential of FC.^3^ Recent systematic reviews (SRs) and meta‐analyses (MAs), however, have failed to establish the superiority of pulpotomy techniques or materials.^4^, ^5^ Overviews of SRs or umbrella reviews have been suggested as a method for evaluating the highest level of evidence and identifying deficiencies therein. Hence, the aims of this study were to: (1) compare the clinical, radiographic, and/or overall success rates of medicaments and techniques; (2) assess the methodological quality of SRs/MAs; and (3) grade the level of evidence for each comparison. Additionally, an attempt was made to identify the sources of ambiguities and develop recommendations for future research.

## 2 MATERIALS AND METHODS

### 2.1 Protocol and registration

An a priori protocol based on the best practices of evidence‐based medicine^6^ and the Joanna Briggs Institute's recommendations for the evaluation of systematic reviews^7^ was developed through an expert‐group discussion and registered in the International Prospective Register of Systematic Reviews (PROSPERO; CRD42021244489). The Preferred Reporting Items for Systematic Reviews and Meta‐Analyses (PRISMA) guidelines were followed in the reporting of this article.^8^

### 2.2 Search strategy

A comprehensive search strategy was developed based upon the PICOS system: Population (P), primary teeth indicated for pulpotomy (due to caries or trauma); Intervention (I), pulpotomy; Comparison (C), other pulp therapies as permitted by study design; Outcomes (O), success rates (overall, clinical, and radiographic); and Study design (S), SR with or without MA, and elements of the research question. PubMed, LILACS, Web of Science, Scopus, Embase, and Cochrane databases were searched on April 4, 2021, without any limitation as to language and year of publication. The detailed search strategy is included in Appendix S1. A search of the gray literature was also performed in Google Scholar and OpenGrey. Two authors (NT and RMJ) performed the literature search independently. A second stage of the literature search included searching of reference lists of the eligible studies and the Cochrane, PROSPERO, and Joanna Briggs Institute's registries of SRs.

### 2.3 Inclusion and exclusion criteria

SRs (1) performed to assess the overall clinical or radiographic efficacy of medicaments or techniques used for pulpotomy of primary teeth, (2) performed by a minimum of two reviewers with literature searches carried out in at least two databases, and (3) with quality analysis of the primary studies performed through a valid and/or reliable tool were included.^9^ Scoping reviews, evidence mapping, narrative reviews, and all forms of primary studies and reviews with unclear methodology in terms of study selection were excluded.^7^

### 2.4 Study selection

Duplicates were removed by means of the EndNote reference management software (EndNote X 8.2; Clarivate Analytics, Philadelphia, PA, USA), and the evaluation of titles and abstracts was performed by two reviewers (NT and SG), with a high level of agreement (Cohen's kappa, 0.93). Full‐text articles were later retrieved, and articles from all the additional sources were compared and added for the final study selection. This was performed by two reviewers (NT and MR), as per the inclusion criteria, with a high level of agreement (Cohen's kappa, 0.87). Any disagreement was resolved by consultation with a third reviewer (VM).

### 2.5 Quality assessment

The quality assessment of the included SRs was performed by “A MeaSurement Tool to Assess systematic Reviews‐2” (AMSTAR‐2),^10^ a tool that helps reviewers ascertain the overall confidence of each of the studies through a checklist of 16 domains. The included SRs were assessed for quality in each of these domains and scored as “yes,” “partially yes,” or “no.” If a SR was found to be deficient in any of the seven domains labeled as critical for the validity of its results, it was considered as “critically low” in quality. Similarly, SRs were also classified as low or high quality, depending upon their acceptability as per AMSTAR‐2.^10^ Further, the risk of bias (ROB) of systematic reviews was assessed by means of the ROBIS tool,^11^ which assesses the ROB through three sections, each evaluating the deficiencies in different components. The analysis of reporting characteristics of these reviews was also performed according to the PRISMA checklist.^8^ The domains of PRISMA, which had been reported in a SR, were scored as 1 and the missing domains as 0. Domains applicable only to the MAs were excluded when SRs without MAs were scored.^9^ This was performed by two reviewers (NT and SS) with a high level of agreement (kappa value ranged from 0.86 to 0.94 for various sections), and any disagreement was resolved by consultation with a third reviewer (AOC).

### 2.6 Data extraction

The data extraction was performed independently by two reviewers (SG and SS) using a specially designed data extraction form, which had been prepiloted in five studies. This form included the SR’s demographic details, review methods, details of the meta‐analyses, and the conclusions. The extracted data were discussed with the team of reviewers to achieve consensus.

### 2.7 Assessment of overlap of primary studies

The overlap of primary studies across the included SRs was performed by creating a citation matrix and estimating the: (1) percentage overlap (%overlap), (2) covered area (CA), and (3) corrected covered area (CCA).^12^ The percentage overlap of the primary studies was calculated by dividing the total number of studies common to at least two SRs by the total number of studies and multiplying by 100. Covered area (CA) was calculated by dividing the total number of publications in each review (including those that had been counted twice) by the multiplication product of the total number of studies and total number of SRs. This number was multiplied by 100 to obtain the covered area (CA). Similarly, the corrected covered area (CCA) was calculated by subtracting the total number of studies (counted once) from the total number of publications included in each review (inclusive of double counting) and dividing it by the number obtained by subtracting the total number of studies from the multiplication product of the total number of studies and the total number of SRs. This was multiplied by 100 to obtain the corrected covered area (CCA).^12^ The overlap was classified as mild (%overlap, <25; CA, <10; and CCA, 0–5), moderate (%overlap, 25–50; CA, 11–25; and CCA, 6–10), high (%overlap, 51–75; CA, 25–40; and CCA, 11–15), or very high (%overlap, >75; CA, >40; and CCA, >16) and was graphically represented by means of a “bubble web diagram.” This was performed by two reviewers (SG and SS) with good agreement (Cohen's kappa ranging from 0.87 to 0.96).

### 2.8 Analyses

The reported success rate of each medicament was derived from different comparisons. As a result, a pool of success rates could be created along with details of the studies that yielded the data. After the exclusion of the studies with reported high risk of bias (ROB) and removal of any duplicate studies, MA was performed to derive the pooled effect size or the success rate of each medicament at different time periods. To compare the efficacy of pulpotomy medicaments and assess the small‐study effects, we used the data from the clinical/radiographic/overall success rates and the total sample size to calculate the summary measures and assess heterogeneity. This was performed by two reviewers (NT and MG) using Stata 16.0. The level of evidence for any comparison was graded according to the criteria adapted from Köhler et al^13^ and modified to suit the present review, through an expert‐group discussion.^14^ The categories were Convincing Evidence (Class I), Highly Suggestive Evidence (Class II), Suggestive Evidence (Class III), Weak Evidence (Class IV), and Negligible Evidence (Class V), and the assessments were done on the basis of sample size, significance of summary associations, evidence of small‐study effects, heterogeneity, and risk of bias in the included primary studies.^15^, ^16^ The risk of bias of the primary studies and sample sizes of more than 100 cases were the modifications to the original criteria (Appendix S2). This was assessed by two reviewers (NT and SS) with good agreement (Cohen's kappa ranging from 0.84 to 0.92). Finally, a summary‐of‐evidence heatmap was created to assess the strength of evidence for the interpretations of a domain (insufficient data/superiority of one material to other/no difference). This was based on the level of evidence measured by the abovementioned Köhler criteria and quality of the source based on the AMSTAR‐2 rating of a SR.

## 3 RESULTS

**FIGURE 1:**
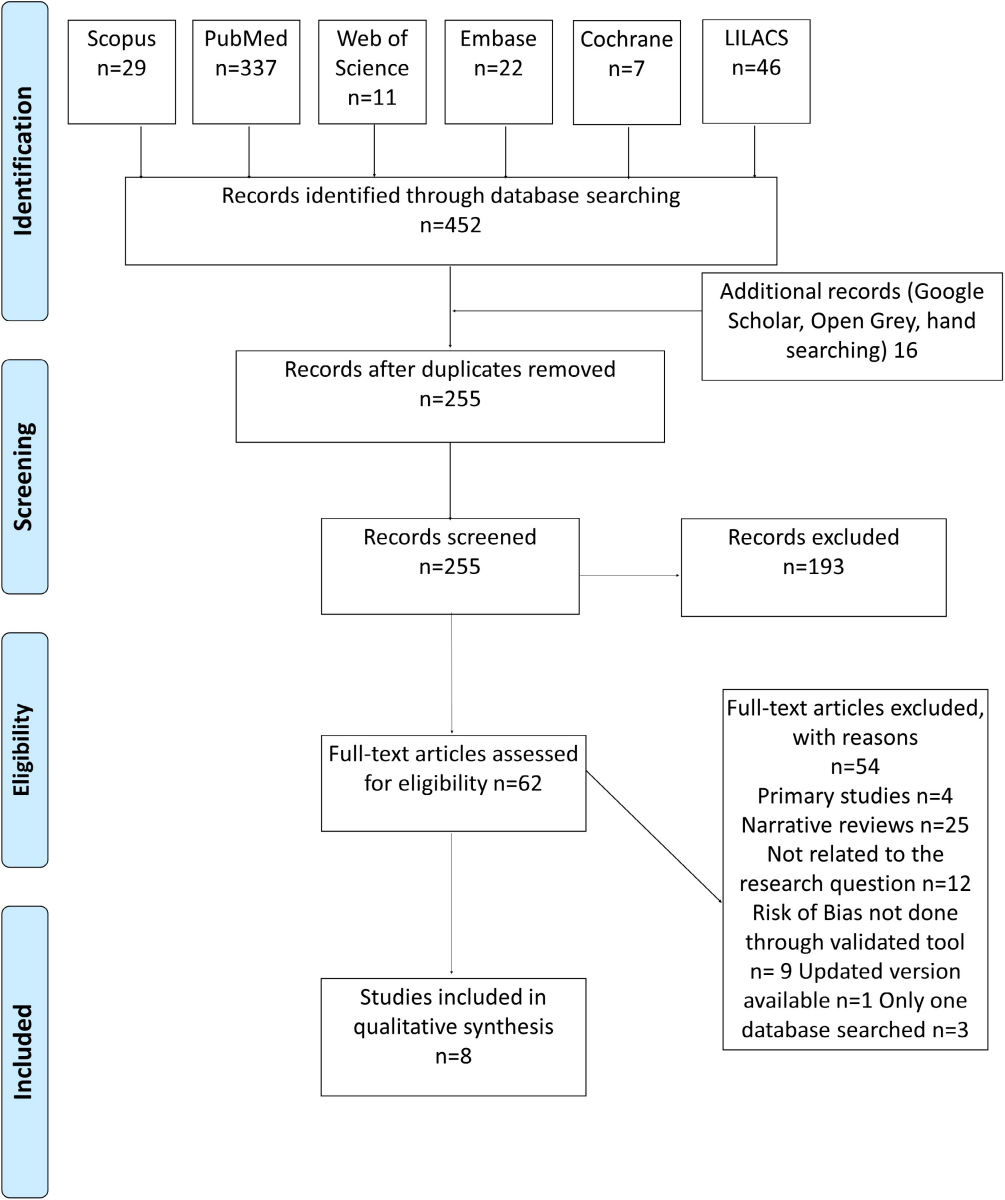
PRISMA chart showing the details of search results and the number of excluded studies along with the reasons for exclusion

The search conducted in the databases resulted in 452 records (Figure 1). After the removal of duplicates, the remaining 255 titles and abstracts were evaluated. Sixteen records were added from other sources, and 62 articles were downloaded for full‐text assessment. Finally, eight SRs were included for qualitative synthesis. The details of excluded papers and reasons for exclusion are presented in Figure 1 and Appendix S3.

### 3.1 Included studies

The included SRs were performed between 2012 and 2020, with only four having a registered a priori protocol (Table 1). The description of the research question of all the reviews revealed a concurrence in their “population” element, along with the inclusion of primary teeth with extensive decay. The “intervention” was variable, with two of them focused on diode lasers (DL) and one each on MTA, ferric sulfate (FS), formocresol (FC), and Biodentine^®^ (BD). Coll et al included all types of vital pulp therapies, whereas Smaïl‐Faugeron et al analyzed all the medicaments used for pulpotomy.^4^, ^5^ The details of the comparators and outcomes are presented in Table 1. The total number of teeth evaluated in any group ranged from 136 to 161 in the study by Marghalani et al,^17^ compared with 28 to 361 teeth in Coll et al^4^, 20 to 518 teeth in Smaïl‐Faugeron et al^5^, 60 to 65 teeth in Nematolahi et al,^18^ 64 to 248 teeth in Junior et al^19^, and 114 to 186 teeth in Jayaraman et al^20^ (Appendix S8).

**TABLE 1:** Details of the included systematic reviews with journal, protocol registration, research question in PICO format, and conclusions

Author	Year	Journal	Protocol Reg.	Aim of the systematic review described in the Population (P), Intervention (I), Comparator (C), and Outcome (O)	Conclusion of the included systematic reviews
De Coster et al (1)	2012	*Int J Ped Dent*	No	**(P)** healthy paediatric patients who required pulpotomy treatment for vital and asymptomatic caries‐affected primary molars, **(I)** laser application prior to dressing/base application, **(C)** other pulpotomy modalities, and **(O)** success rate after a minimum of 6 months	Laser is less successful than conventional pulpotomy techniques. General recommendations for the clinical use of lasers in pulpotomy in primary teeth cannot yet be formulated.
Marghalani et al (2)	2014	*J Am Dent Assoc*	No	**(P)** healthy paediatric patients who required pulpotomy treatment for vital and asymptomatic caries‐affected primary molars, **(I)** primary molars treated with MTA as a pulpotomy dressing material, **(C)** primary molars treated with FC, and **(O)** clinical and radiographic success rates after an observation period of at least 24 months	Pulpotomy procedures performed in primary molars involving the use of MTA or FC showed comparable clinical success rates.
Coll et al (3)	2017	*Ped Dent*	PROSPERO 2015: CRD42015006942	**(P)** primary teeth with deep caries lesions **(I)** indirect pulp therapy (IPT), direct pulp capping (DPC), and pulpotomy; **(C)** one VPT with another; and **(O)** clinical and radiographic outcome after a minimum of 12 months	The highest level of success and quality of evidence supported IPT and the pulpotomy techniques of MTA and FC for the treatment of deep caries in primary teeth after 24 months. DPC showed success rates similar to those of IPT and MTA or FC pulpotomy, but the quality of the evidence was lower.
Nematollahi et al (4)	2018	*Eur Arch Paediatr* Dent	No	**(P)** primary teeth, **(I)** laser pulpotomy, **(C)** other pulpotomy modalities, and **(O)** clinical and radiographic success rates	For primary molar pulpotomy, the laser technique showed clinical and radiographic results comparable with those of other conventional pulpotomy medicaments, including formocresol and MTA.
Nuvvula et al (5)	2018	*Eur Arch Paediatr Dent*	PROSPERO 2017:CRD42017065357	**(P)** primary molars indicated for pulpotomy, **(I)** ferric sulfate pulpotomy, **(C)** other pulpotomy materials, and **(O)** success and failure	Limited evidence of high‐quality studies in the existing English literature to support FS as an effective alternative compared with other pulpotomy medicaments in primary molars
Smaïl‐Faugeron et al (6)	2018	*Cochrane Database Syst Rev*	Yes, Cochrane	**(P)** children with extensive decay in primary teeth, **(I)** pulpotomy with one type of medicament, **(C)** pulpotomy with alternative medicament or different techniques, and **(O)** overall, clinical, and radiographic success rates	MTA may be the most efficacious medicament for pulpotomy of primary teeth. Future research could be undertaken to confirm whether Biodentine, enamel matrix derivative, laser treatment, and Ankaferd Blood Stopper are acceptable second choices, and whether, where none of these treatments can be used, application of sodium hypochlorite is the safest option. Formocresol, though effective, has known concerns about toxicity.
Stringhini Junior et al (7)	2019	*Clin Oral Investig*	No	**(P)** primary teeth of children with deep caries lesions, **(I)** Biodentine pulpotomy, **(C)** MTA pulpotomy, and **(O)** clinical and radiographic success rates	There is no superiority of one material to the other, MTA versus Biodentine.
Jayaraman et al (8)	2020	*Quintessence Int*	PROSPERO (CRD42018090044)	**(P)** primary teeth of children with deep caries lesions, **(I)** formocresol pulpotomy, **(C)** ferric sulfate pulpotomy, and **(O)** clinical and radiographic success rates after 24 months	Formocresol and ferric sulfate show comparable clinical and radiographic success rates as pulpotomy material in primary molars at 24 months based on the studies with low‐to‐moderate quality of evidence

Abbreviations: FC, formocresol; MTA, mineral trioxide aggregate.

### 3.2 Quality assessment

**FIGURE 2:**
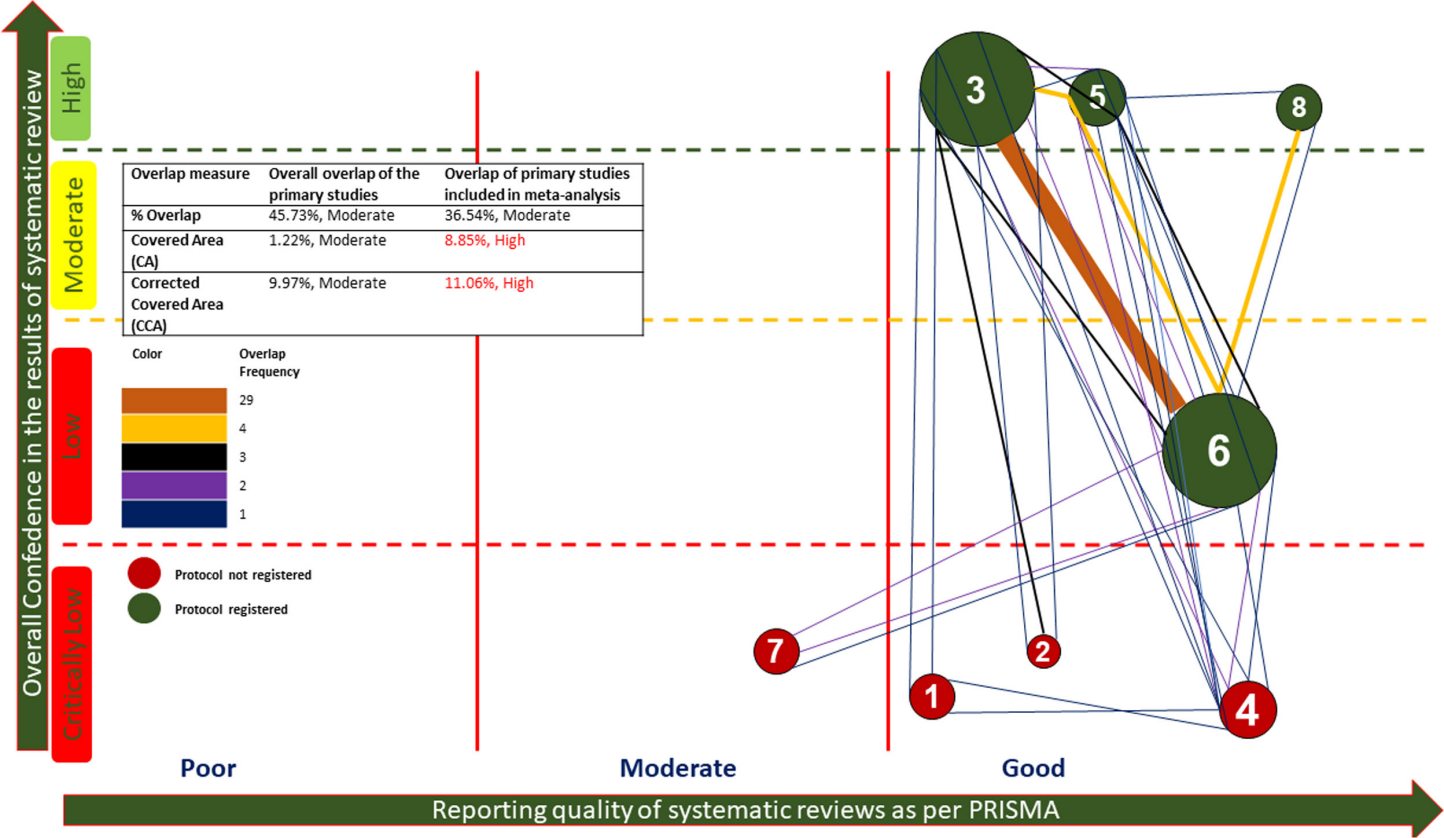
Bubble web diagram showing the overlap of primary studies along with the quality of systematic reviews as per AMSTAR‐2, reporting characteristics as per PRISMA. The size of the bubble corresponds to the number of included studies. Color denotes the protocol registration, with a number identifying the individual review (1—De Coster et al; 2—Marghalani et al; 3—Coll et al; 4—Nematollahi et al; 5—Nuvvula et al; 6—Smaïl‐Faugeron et al; 7—Stringhini Junior et al; and 8—Jayaraman et al)

The quality of four of the reviews as per AMSTAR‐2 was found to be critically low,^17^, ^18^, ^19^, ^21^ due to the absence of an a priori protocol and lack of assessment of publication bias (Figure 2, Appendix S4). In one review, only the publication bias was not assessed, and it was rated low in confidence.^5^ Three of the included SRs were found to have high confidence^4^, ^20^, ^22^ (Figure 2, Appendix S4). As per ROBIS, the risk of bias was found to be high in five and low in three of SRs. The major deficiencies were related to the interpretation of findings addressing the concerns and confounding factors, whereas other aspects had been better addressed (Appendix S9). Reporting characteristics were found to be good (>75%) in all the reviews except by Stringhini Junior et al^19^ (Figure 2, Appendix S5), which had deficiencies in 14 areas expected as per PRISMA. These included lack of search strategy details, details of synthesis methods, reporting bias, details of the excluded studies, a priori protocol, and sources of support. The details of the search methods, inclusion and exclusion criteria, and ROB analysis of the included SRs are summarized in Table 2.

**TABLE 2:** Details of the included systematic reviews with type of study included, exclusion criteria, databases searched, limitations in search, gray literature and reference searching, tool used for risk‐of‐bias analysis, and GRADE of evidence

Author (first author)	Year	Type of study included/criteria defined and exclusion criteria	Databases searched	Limitations in search	Gray Literature and reference searching	Tool used for risk‐of‐bias analysis	GRADE of evidence
De Coster et al	2012	Randomized controlled trials (RCT), case–control studies, or case series (CS) involving at least ten teeth. Both clinical and radiographic outcome measures. At least 6‐month follow‐up. Exclusion criteria not mentioned.	MEDLINE, Web of Science, and Cochrane	None	Yes/Yes	Dutch Cochrane Collaboration risk‐of‐bias assessment tool	Oxford Centre for Evidence‐Based Medicine guidelines
Marghalani et al	2014	NM	MEDLINE, Web of Science, and Cochrane Central Register of Controlled Trials	January 1, 1990, through May 9, 2013	No	Cochrane Collaboration's risk‐of‐bias assessment tool	No
Coll et al	2017	Studies with a minimum of 12 months’ follow‐up. Those with non‐carious pulp exposure were excluded.	MEDLINE, Embase, Cochrane Central Register of Controlled Trials (CENTRAL), EBSCO—Dentistry and Oral Sciences Source, ICTRP (trials database)	None	Yes/Yes	Cochrane Collaboration's risk‐of‐bias assessment tool	GRADE
Nematollahi et al	2018	Original clinical trials (only for meta‐analysis) and at least 6 months of follow‐up (only for meta‐analysis); exclusion criteria, NM	PubMed, Scopus, EMBASE, Cochrane, and ISI Web of Knowledge	None	No	Jadad et al (1996)	NM
Nuvvula et al	2018	Randomized clinical trials, follow‐up time of at least 6 months. Both clinical and radiographic criteria. Animal studies, case reports, observational studies, and single‐arm clinical trials. Review articles excluded.	PubMed, EBSCOhost, Cochrane Library, and ProQuest	November 1991 to January 2017, English language	No/Yes	Fuks and Papagiannoulis (2006)	NM
Smaïl‐Faugeron et al	2018	RCT, English‐language follow‐up period of at least 6 months. Non‐RCT, abstracts only, insufficient information, and attempts to contact the authors were unsuccessful, studies without comparison of biomaterials, animal studies, in vitro studies, reviews, duplicates, restorative dentistry, ongoing trials terminated because the use of one of the materials was discontinued, case reports excluded	Cochrane Oral Health's Trials Register, Cochrane Central Register of Controlled Trials, MEDLINE, Embase, and Web of Science	None	Yes/Yes	Cochrane Collaboration's risk‐of‐bias assessment tool	GRADE
Stringhini Junior et al	2019	Clinical studies with a minimum of 6 months’ follow‐up. Case reports, reviews, in vitro studies, and the studies that did not focus on Biodentine or MTA were excluded.	PubMed, Lilacs, Cochrane, Embase, Scopus, Web of Science, CINAHL, EBSCO, and clinical trials	None	No/Yes	Cochrane Collaboration's risk‐of‐bias assessment tool	NM
Jayaraman et al	2020	Randomized clinical trials with more than 6 months’ follow‐up were included. Non‐randomized clinical trials, case reports, case series, and reviews were excluded.	PubMed, Scopus, and EBSCOhost	English language	No/Yes	Cochrane Collaboration's risk‐of‐bias assessment tool	GRADE

Abbreviation: NM, not mentioned.

### 3.3 Overlap of the primary studies

The overlap of primary studies is diagrammatically represented in Figure 2, with the maximum number of overlaps (*n* = 29) seen between the reviews by Coll et al and Smaïl‐Faugeron et al^4^, ^5^ (Figure 2). The quantitative assessment revealed an overall moderate overlap (%overlap, 45.73%; CA, 1.22%; and CCA, 9.97%).^12^ This was found to be high as per the CA and CCA in the studies included in the MAs (Figure 2, Appendix S6).

### 3.4 Analyses of the qualitative outcomes

In only two of the included SRs were MAs not performed.^21^, ^22^ De Coster et al (2012) found DL to be less successful than conventional pulpotomy techniques and highlighted the paucity of good‐quality studies.^21^ Six years later, Nematollahi et al showed comparable clinical and radiographic success rates of this technique with other conventional pulpotomy medicaments.^18^ Marghalani et al observed the equal efficacy of MTA or FC (Table 1).^17^ Similarly, Coll et al stated that the highest level of success with the best‐quality evidence supported MTA and FC as pulpotomy agents for the treatment of deep caries in primary teeth.^4^ Nuvvula et al stated that FS was an effective alternative to other pulpotomy medicaments in primary molars, though high‐quality studies were limited.^22^ A Cochrane review carried out by Smaïl‐Faugeron et al highlighted that MTA may be the most efficacious medicament for pulpotomy, with limited evidence to confirm the efficacy of other medicaments such as BD, enamel matrix derivative (EMD), DL, and Ankaferd Blood Stopper (ABS) as acceptable second choices.^5^ Similar findings were also reported in the recent SRs by Stringhini Junior et al and Jayaraman et al, which did not find one material to be superior to the other (Table 1).^19^, ^20^

### 3.5 Analyses of the quantitative outcomes

This review attempted to analyze 151 MAs, which had been presented in the included studies. All the authors had used random‐effects models for assessing the clinical/radiographic/overall success rates, except Smaïl‐Faugeron et al, who used a fixed‐effects model and compared the failures.^5^ The risk ratio (RR) was the preferred summary measure used by the authors except for Nematollahi et al, who used odds ratios (ORs) for presenting the comparisons between clinical and radiographic success rates at 6–30 months (Appendix S7).^18^ To ensure the uniformity of comparisons, we utilized the data from primary studies included in all the MAs for calculating the summary measures, along with the heterogeneity, 95% confidence intervals of the measures, and the small‐study effect (funnel plot with Egger's test) (Appendix S7).

The maximum number of primary studies (*n* = 13) and the highest sample size were seen in the comparison of MTA vs full‐strength/1:5‐dilution FC for their clinical success rates at 6 months (MTA, 518; FC, 530), whereas the lowest (*n* = 25/group) was seen in comparisons of electrosurgery (ES) vs DL and ES vs FS.^5^ The data from only one study were used for comparing MTA with DL (clinical success rate at 6–30 months), BD with DL (clinical and radiographic success rates at 6–30 months), and CH with DL (radiographic success rate at 6–30 months).^18^ Fifty meta‐analyses were based on the data from two studies each.^4^, ^5^, ^18^ Further, it was observed that 95 MAs included one or more studies with high ROB,^5^, ^17^, ^18^, ^19^, ^20^ whereas 51 included studies with moderate/unclear ROB.^4^, ^5^, ^18^ Only five of the comparisons utilized the data exclusively from studies with low ROB (Appendix S7).

**FIGURE 3:**
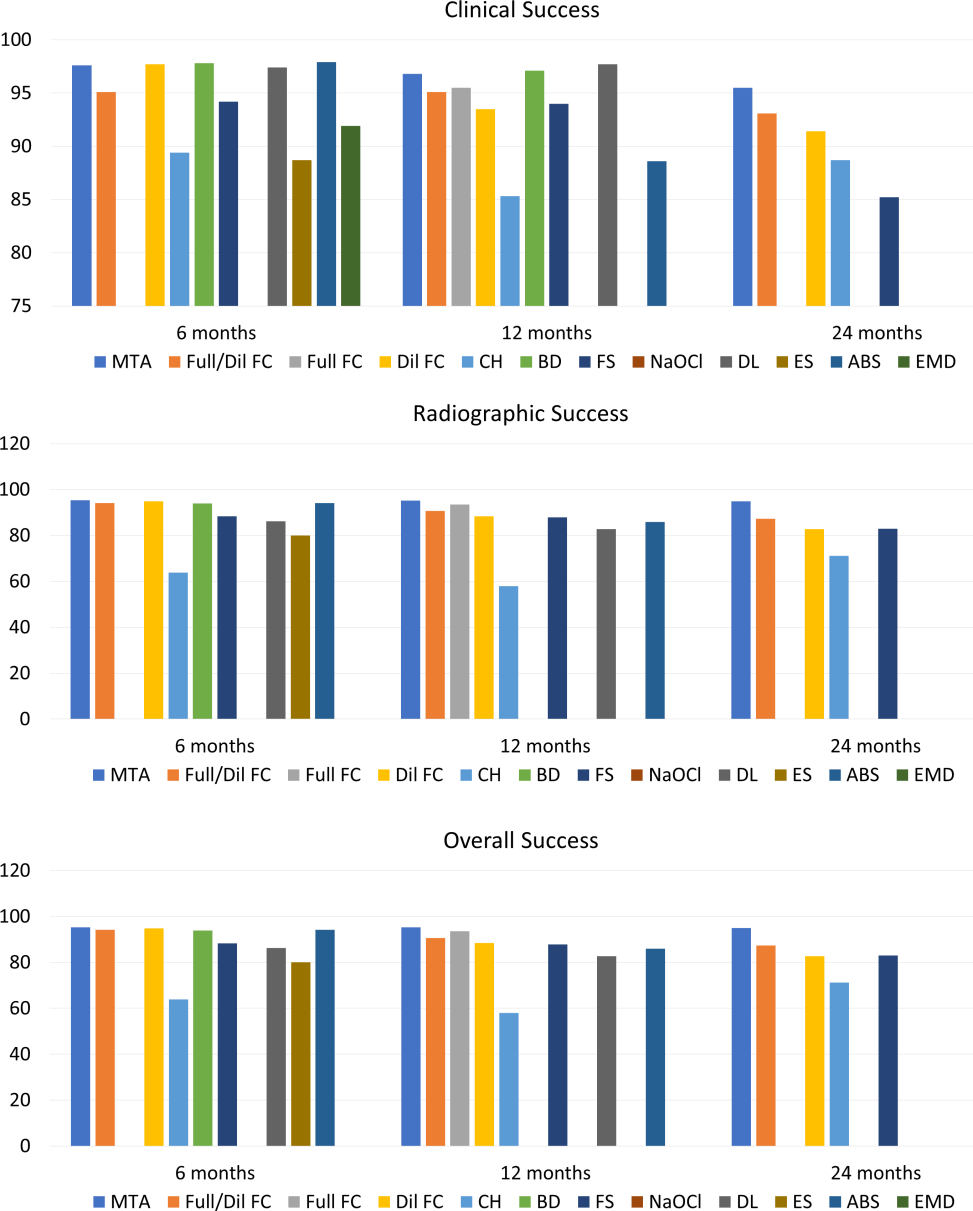
Details of the clinical, radiographic, and overall success rates of different medicaments/techniques (MTA—mineral trioxide aggregate; Full/Dil FC—full‐strength/1:5 diluted formocresol; Full FC—full‐strength formocresol; Dil FC—diluted formocresol; CH—calcium hydroxide; BD—Biodentine; FS—ferric sulfate; NaOCl—sodium hypochlorite; DL—diode laser; ES—electrosurgery; ABS—Ankaferd Blood Stopper; and EMD—enamel matrix derivative)

The success rates of the pulpotomy medicaments/techniques were also assessed in 82 MAs of data pooled from the studies with unclear or low ROB. Clinical success rates were found to be above 90% after six months in all the medicaments except CH (89.4%) and ES (88.7%) (Figure 3, Appendix S8). This trend continued for clinical success rates at 12 months, with CH showing the lowest rate (85.3%). The 24‐month data were available for only five medicaments (MTA, Full/Dil FC, Dil FC, CH, and FS), with FS showing the least clinical success (80.4%). The radiographic success rate was found to be above 95% for MTA at 6, 12, and 24 months, whereas the lowest was seen with CH at 12 months (57.9%). The overall success rates of all of these at the three time periods were observed to be above 80%, with the highest seen in MTA (6, 12, and 24 months) and the lowest in CH (6 months, 63.2%; 12 months, 64.7%; and 24 months, 43%). The long‐term evaluations (12, 24 months) were found to be limited for several medicaments, due to either the lack of data or the paucity of low/unclear risk studies (Figure 3, Appendix S8).

Most comparisons did not exhibit superiority of any one medicament/technique. MTA, however, was found to be superior to full‐strength/1:5‐dilution FC and full‐strength FC in terms of the radiographic success rate at 24 months (RR, 1.05, 95% CI = 1.00–1.10, *p* < .05; and RR, 1.09, 95% CI = 1.03–1.15, *p* = .003, respectively)^5^ (Appendix S7). Similarly, MTA was also found to be better than CH in terms of its radiographic and overall success rates at 12 months (radiographic: RR, 1.44, 95% CI = 1.20–1.71, *p* < .0001^5^; overall: RR, 1.50, 95% CI = 1.28–1.76, *p* < .00001)^4^ and 24 months (radiographic: RR, 1.73, 95% CI = 1.29–2.31, *p* = .0002^5^; overall: RR, 1.96, 95% CI = 1.52–2.53, *p* < .00001,^4^ and RR, 1.37, 95% CI = 1.04–1.81, *p* = .03)^5^ (Appendix S7). Other comparisons that showed differences were FC vs CH for radiographic success rates at 12 months (RR, 0.70, 95% CI, 0.49–1, *p* < .05)^5^ and overall success rates at 12 months (CH vs FC: RR, 1.43, 95% CI, 1.14–1.79, *p* = .002),^4^ 18 months (CH vs FC: RR, 1.25, 95% CI, 1.02–1.53, *p* = .03),^4^ and 24 months (FC vs CH: RR, 0.77, 95% CI, 0.64–0.93, *p* = .008)^5^; CH vs FC (RR, 1.76, 95% CI, 1.40–2.23, *p* < .00001)^4^; CH vs FS for overall success at 24 months (RR, 1.57, 95% CI, 1.19–2.06, *p* <.001)^4^; and sodium hypochlorite (NaOCl) vs FC for overall success at 18 months (RR, 0.83, 95% CI, 0.72–0.96, *p* <.01).^4^ The heterogeneity in the majority of comparisons (*I* ^2^ values) was low (≤25%) (*n* = 107).^4^, ^5^, ^17^, ^18^, ^19^, ^20^ The small‐study effect was absent in all comparisons except MTA vs FS for overall success rate at 6 months^5^ and 12 months (Appendix S8).^4^

### 3.6 Summary of evidence

**FIGURE 4:**
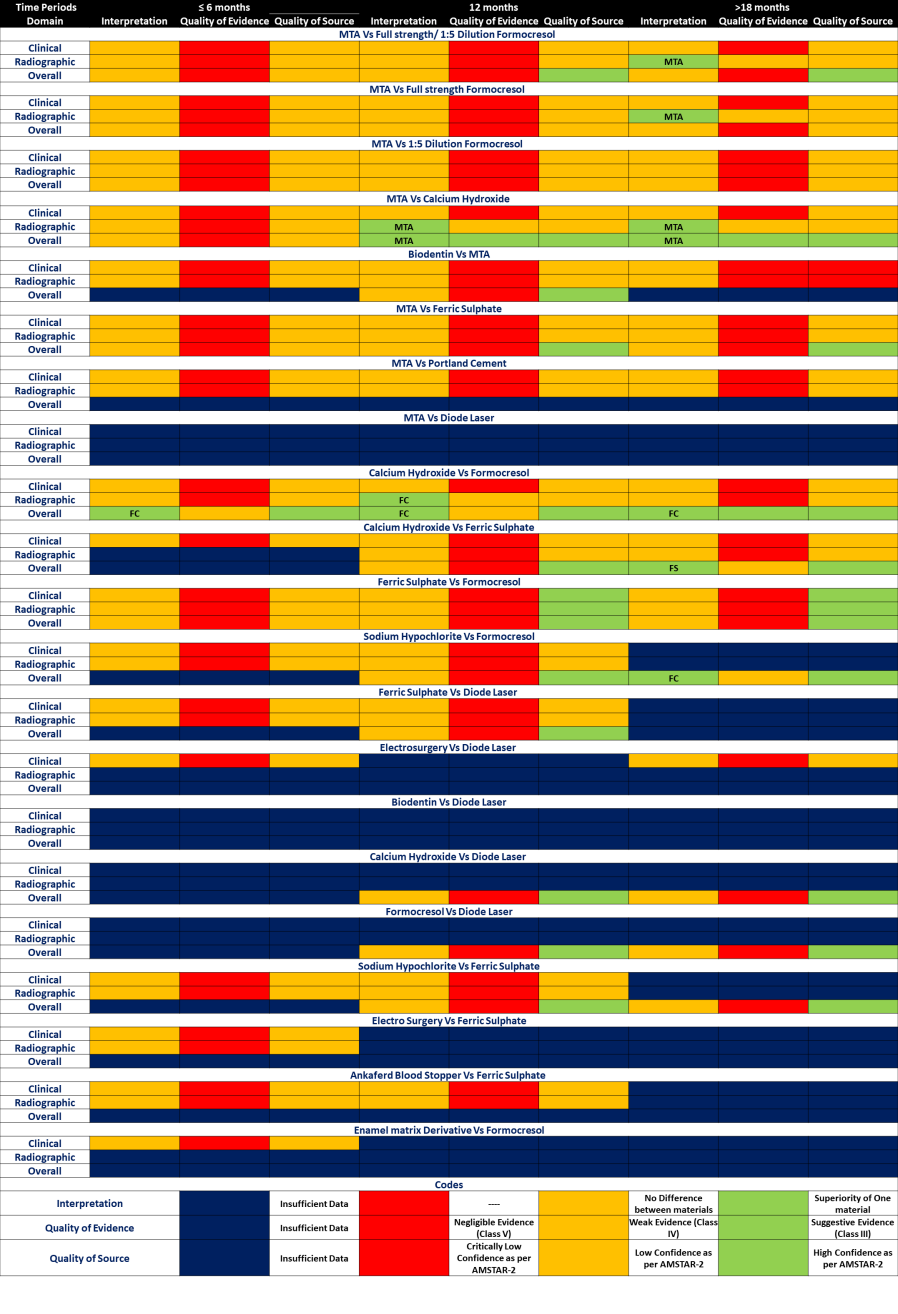
Summary‐of‐evidence diagram showing the interpretations of the meta‐analyses related to comparisons of different medicaments/techniques for various domains and time periods, exhibiting their level of evidence and the quality of the source systematic review as per AMSTAR‐2

The summary‐of‐evidence heatmap represented 63 domains and 189 interpretations. Among these, 12 showed a clear superiority of one medicament to the other. MTA showed superiority to full‐strength/1:5‐dilution and full‐strength FC with weak evidence and a low‐quality SR source. It also showed better radiographic and overall success rates than CH at 12 and 24 months. The quality of evidence was weak, and the quality of the SR source was low for radiographic success, whereas the overall success rate at both time periods showed suggestive evidence and high‐quality SR sources. The other medicaments that showed significant superiority in comparisons were FC and FS. FC was found to be better than CH for radiographic success rate at 12 months, with weak evidence and low‐quality sources, along with the overall success rates at 6 and 12 months with weak evidence and high‐quality sources. Similar findings were also observed for FC’s significant overall success rates over NaOCl and superior overall success rates of FS over CH at 24 months. The interpretation of FC’s better success rate in the overall domain at 24 months exhibited suggestive evidence and high‐quality sources (Figure 4, Appendix S7). There were 25 domains (39.7%) of 6 months, 21 (33.3%) of 12 months, and 30 (47.6%) of periods greater than 18 months, which showed gaps in the data.

## 4 DISCUSSION

Overviews of SRs are regarded as an excellent source of evidence analysis and knowledge bases for any domain of a specialty.^7^, ^14^, ^23^ They also serve as an exercise for identifying the gaps in both primary and secondary researches, which can ultimately guide future research toward high‐quality evidence and its analysis.^23^, ^24^, ^25^ This overview was successful in presenting the success rates and summarizing the available comparisons for medicaments and techniques used for pulpotomy of primary teeth with pulp exposure. By most of the materials/techniques, pulpotomy showed success rates above 80% in all comparisons, making it a very effective, vital pulp therapy procedure for primary teeth. CH showed considerably lower success rates in all categories, which is a concern. Hence, its use must be discouraged and replaced by other alternatives identified in this review. Further, we could identify only 12 of 189 comparisons (6.35%) that could show a definitive superiority of a medicament or technique. Lack of data was higher in the periods greater than 18 months and was related to the newer medicaments and techniques.

In 2018, Wormland and Evans stated that SRs and MAs, though considered as the highest level of evidence, can convey only the findings that the primary studies report, and hence, their conclusions depend largely on the heterogeneity of study designs and their reporting.^26^ Quality of the majority of primary studies reported in the included SRs was poor or moderate/unclear. This was more evident in the MAs, 62.9% of which had included the data from one or more studies with high ROB. The best summaries can be derived from the adequate number of homogenous primary studies.^25^ This was another lack observed in the included MAs, because three of them had been based on one and 31% had been based upon only two primary studies. The lack of primary studies in an area is also an opportunity for researchers to add to the existing knowledge through well‐designed studies.

Though 87.5% of SRs had been reported as per the recommended PRISMA guidelines, their quality was low or critically low, and ROB was high in five of the eight SRs (62.5%). This can be attributed to certain common methodological limitations such as the lack of an a priori protocol, lack of assessment of publication bias, and interpretation of findings addressing concerns and confounding factors. If we evaluate the conclusions of SRs on the basis of their confidence, only the recommendations of Coll et al for MTA and FC, Nuvvula et al for FS being comparable with other agents, and Jayaraman et al for comparable success of FC and FS can be viewed with high confidence. In the presence of the quantitative data, however, the results of meta‐analyses are more reliable. Apart from the summary measures, the sample size, heterogeneity reflected by *I* ^2^, the p‐value of the comparison, and the 95% confidence interval and small‐study effects of the summary measure are all contributory to the final assertions.^4^, ^18^, ^19^ The evidence in the outcomes was graded as per the criteria given by Köhler et al After suitable modification, its content and face validity were also established.^11^ On the basis of these criteria, only 12 comparisons showed suggestive or weak evidence. The lack of evidence in recommendations regarding the choice of pulpotomy agents is a major limitation identified by our review.

This overview is one of the very few works that have assessed the overlap of primary studies in the included SR and MAs. In 2021, Solmi et al stated that the overviews of SRs have an overlap that may result in the exaggeration of the qualitative conclusions and outcomes of meta‐synthesis, if performed.^27^ In 2014, Pieper et al suggested that the overlap of the studies in an overview can be evaluated by three methods via the creation of a citation matrix.^12^ The simplest method is to calculate the %overlap by counting the total number of overlaps and dividing them by the total number of studies. The more reliable methods are covered area and corrected covered area, which are based upon the rows and columns of the citation matrix and the overlaps observed.^12^ This was calculated in the present study so that the overlap between the included SRs and MAs could be assessed. It was found to be moderate for SRs, whereas MAs exhibited a high grade of overlap. The graphical representation of this characteristic also reflected a more mesh network of overlaps between SRs. Since six of these reviews had been done within a span of three years (2017–2020), and four had not been registered and had similar research questions, the overlap was understandable.

The limitations of this overview of SRs include the moderate to high grade of overlap of primary studies between the included reviews and subjectivity in the nature of the literature search, inclusion, quality analysis, and analysis of the results. The possibilities of bias in these steps were minimized by having a diverse and experienced research group, development of the a priori protocol and search strategy through expert‐group discussion, having two experienced reviewers with good agreement (measured by Cohen's kappa) for each step, and assessment of the quality of evidence through multiple parameters.^6^, ^7^, ^26^

Pulp biology is an evolving science and will undoubtedly undergo numerous innovations for better success rates of vital pulp therapies and pulpotomy.^2^ Clinical research and the resulting evidence syntheses will require the following recommendations to overcome the existing gaps in knowledge:

1. **Uniformity of study designs and their high quality:** Future studies must be designed as randomized control trials and be uniform in their design in terms of sampling, case selection, treatment protocol, outcome assessment, the follow‐up protocol, and reporting. This can overcome several of the biases prevalent in non‐randomized studies. The recommendations for case selection for pulpotomy and the clinical procedures play an important role in treatment success and must be ensured by prior training and calibration of the operators.
2. **Identification of future focus areas:** The areas or comparisons identified as lacking evidence or based upon fewer or lower‐quality primary studies must be prioritized by the primary and EBM researchers. This will lead to the development of a better knowledge base, especially with newer materials and extended time periods, and increase the reliability of future SRs in this area.^9^
3. **Improving the quality of SRs and MAs:** Future systematic reviews must be based upon the recommendations of the Cochrane Handbook and PRISMA.^6^, ^8^ This requires an a priori registration in the SR registries such as PROSPERO, JBI, Cochrane, and Open Science Framework. Additionally, the reporting of publication bias must be performed for each comparison so that the small‐study effect can be assessed. The analysis of the requirements of the quality analysis tools of SRs, such as AMSTAR‐2, also helps reviewers address critical areas and improve quality.^10^

The pulpotomy medicaments/techniques, except CH, had success rates of more than 80% for all domains and time periods. Most comparisons did not report any differences in clinical, radiographic, or overall success rates. MTA, however, was found to have better radiographic and overall success rates than CH at periods greater than 12 and 18 months. It also had greater radiographic success than full‐strength/1:5 diluted and full‐strength FC at 24 months. FC was found to have better overall success rates than CH at all time periods and better radiographic success rates at 12 months. FS was also found to be better than CH after 18 months. Four of the included SRs were found to be critically low, whereas one was low in confidence. The analysis of the evidence could establish only 12 comparisons with suggestive or weak evidence, whereas all others had either negligible evidence or insufficient data. This study highlights the lack of evidence regarding the choice of pulpotomy agents for caries‐affected primary teeth and elucidates the domains that require primary studies in the future.

## CONFLICT OF INTEREST

The authors declare no conflicts of interest.

## AUTHOR CONTRIBUTION

All authors made substantive contributions to this study and have reviewed the final manuscript prior to its submission.

## Supporting information

Supplementary Material

## Data Availability

The data that supports the findings of this study are available in the supplementary material of this article.
