# Supplementary material for: Success of medicaments and techniques for pulpotomy of primary teeth: An overview of systematic reviews
Source: Int J Paediatr Dent. 2022 Apr 26;32(6):828–42. doi: 10.1111/ipd.12963 (PMC9790730; doi:10.1111/ipd.12963)
Supplement: Supplementary file 1 — Supplementary Material [file IPD-32-828-s001.docx]

**Title: Success of medicaments and techniques for pulpotomy of primary teeth: an overview of systematic reviews**

**Legends for the supplementary files**

**Appendix 1**: Search Strategy for different databases and their results

**Appendix 2**: Proposed criteria for classification of the evidence of the outcomes of the meta-analysis of intervention studies in dental research

**Appendix 3**: List of the excluded studies with reasons for exclusion

**Appendix 4**: The overall quality of the included systematic reviews using AMSTAR-2

**Appendix 5**: Details of the reporting characteristics of the included systematic reviews

**Appendix 6**: Details of the primary studies included in the systematic reviews along-with their overlap

**Appendix 7**: Details of the included meta-analyses for different comparisons along with the small study effect and level of evidence

**Appendix 8**: Details of the pooled effect sizes of clinical, radiographic and overall success of different pulpotomy medicaments/techniques at different time periods (6 months, 12 months, 18 months, 24 months).

**Appendix 9:** Risk of bias in the included systematic reviews as assessed by ROBIS tool.

**Appendix 1: Search Strategy for different databases and their results**

|  | Search Strategy | The search was conducted by creating three fields: 1- Pulpotomy OR Pulp-therapy OR Vital-Pulp-Therapy OR PT OR Apexogenesis OR Laser OR MTA OR Mineral-Trioxide-Aggregate OR Calcium-Hydroxide OR Biodentine OR Formocresol OR Electrosurgical OR Calcium-Enriched-Mixture OR Simvastatin OR Zinc-Oxide-Eugenol OR Sodium-Hypochlorite; 2- Primary-tooth OR Milk-Tooth OR Temporary-tooth OR deciduous-tooth; 3- Systematic-Review OR Review OR Meta-analysis. These were later combined by using Boolean operator “AND” with suitable modifications for different databases. |
| --- | --- | --- |
| Database | Search Results | Search Strategy |
| PubMed | 337 | ("pulpotomy"[MeSH Terms] OR "pulpotomy"[All Fields] OR "pulpotomies"[All Fields] OR (("dental pulp"[MeSH Terms] OR ("dental"[All Fields] AND "pulp"[All Fields]) OR "dental pulp"[All Fields] OR "pulp"[All Fields]) AND ("therapeutics"[MeSH Terms] OR "therapeutics"[All Fields] OR "therapies"[All Fields] OR "therapy"[MeSH Subheading] OR "therapy"[All Fields] OR "therapy s"[All Fields] OR "therapys"[All Fields])) OR (("vital signs"[MeSH Terms] OR ("vital"[All Fields] AND "signs"[All Fields]) OR "vital signs"[All Fields] OR "vital"[All Fields] OR "vitally"[All Fields] OR "vitals"[All Fields]) AND ("dental pulp"[MeSH Terms] OR ("dental"[All Fields] AND "pulp"[All Fields]) OR "dental pulp"[All Fields] OR "pulp"[All Fields]) AND ("therapeutics"[MeSH Terms] OR "therapeutics"[All Fields] OR "therapies"[All Fields] OR "therapy"[MeSH Subheading] OR "therapy"[All Fields] OR "therapy s"[All Fields] OR "therapys"[All Fields])) OR "PT"[All Fields] OR ("apexification"[MeSH Terms] OR "apexification"[All Fields] OR "apexogenesis"[All Fields]) OR ("laser s"[All Fields] OR "lasers"[MeSH Terms] OR "lasers"[All Fields] OR "laser"[All Fields] OR "lasered"[All Fields] OR "lasering"[All Fields]) OR "MTA"[All Fields] OR ("mineral trioxide aggregate"[Supplementary Concept] OR "mineral trioxide aggregate"[All Fields]) OR ("calcium hydroxide"[MeSH Terms] OR ("calcium"[All Fields] AND "hydroxide"[All Fields]) OR "calcium hydroxide"[All Fields]) OR ("tricalcium silicate"[Supplementary Concept] OR "tricalcium silicate"[All Fields] OR "biodentine"[All Fields]) OR ("formocresol"[Supplementary Concept] OR "formocresol"[All Fields]) OR (("electrosurgical"[All Fields] OR "electrosurgically"[All Fields]) AND ("calcium"[MeSH Terms] OR "calcium"[All Fields] OR "calciums"[All Fields] OR "calcium s"[All Fields]) AND ("enrich"[All Fields] OR "enriched"[All Fields] OR "enriches"[All Fields] OR "enriching"[All Fields] OR "enrichment"[All Fields] OR "enrichments"[All Fields]) AND ("mixture"[All Fields] OR "mixture s"[All Fields] OR "mixtures"[All Fields])) OR ("simvastatin"[MeSH Terms] OR "simvastatin"[All Fields] OR "simvastatin s"[All Fields] OR "simvastatins"[All Fields]) OR ("zinc oxide eugenol cement"[MeSH Terms] OR ("zinc"[All Fields] AND "oxide eugenol"[All Fields] AND "cement"[All Fields]) OR "zinc oxide eugenol cement"[All Fields] OR ("zinc"[All Fields] AND "oxide"[All Fields] AND "eugenol"[All Fields]) OR "zinc oxide eugenol"[All Fields]) OR ("sodium hypochlorite"[MeSH Terms] OR ("sodium"[All Fields] AND "hypochlorite"[All Fields]) OR "sodium hypochlorite"[All Fields])) AND ("tooth, deciduous"[MeSH Terms] OR ("tooth"[All Fields] AND "deciduous"[All Fields]) OR "deciduous tooth"[All Fields] OR ("primary"[All Fields] AND "tooth"[All Fields]) OR "primary tooth"[All Fields] OR ("tooth, deciduous"[MeSH Terms] OR ("tooth"[All Fields] AND "deciduous"[All Fields]) OR "deciduous tooth"[All Fields] OR ("milk"[All Fields] AND "tooth"[All Fields]) OR "milk tooth"[All Fields]) OR ("tooth, deciduous"[MeSH Terms] OR ("tooth"[All Fields] AND "deciduous"[All Fields]) OR "deciduous tooth"[All Fields] OR ("deciduous"[All Fields] AND "tooth"[All Fields])) OR (("temporaries"[All Fields] OR "temporary"[All Fields]) AND ("teeth s"[All Fields] OR "teeths"[All Fields] OR "tooth"[MeSH Terms] OR "tooth"[All Fields] OR "teeth"[All Fields] OR "tooth s"[All Fields] OR "tooths"[All Fields]))) AND ("systematic review"[Publication Type] OR "systematic reviews as topic"[MeSH Terms] OR "systematic review"[All Fields] OR ("review"[Publication Type] OR "review literature as topic"[MeSH Terms] OR "review"[All Fields]) OR ("meta analysis"[Publication Type] OR "meta analysis as topic"[MeSH Terms] OR "meta analysis"[All Fields]))  Translations  Pulpotomy: "pulpotomy"[MeSH Terms] OR "pulpotomy"[All Fields] OR "pulpotomies"[All Fields]  Pulp: "dental pulp"[MeSH Terms] OR ("dental"[All Fields] AND "pulp"[All Fields]) OR "dental pulp"[All Fields] OR "pulp"[All Fields]  therapy: "therapeutics"[MeSH Terms] OR "therapeutics"[All Fields] OR "therapies"[All Fields] OR "therapy"[Subheading] OR "therapy"[All Fields] OR "therapy's"[All Fields] OR "therapys"[All Fields]  Vital: "vital signs"[MeSH Terms] OR ("vital"[All Fields] AND "signs"[All Fields]) OR "vital signs"[All Fields] OR "vital"[All Fields] OR "vitally"[All Fields] OR "vitals"[All Fields]  Pulp: "dental pulp"[MeSH Terms] OR ("dental"[All Fields] AND "pulp"[All Fields]) OR "dental pulp"[All Fields] OR "pulp"[All Fields]  Therapy: "therapeutics"[MeSH Terms] OR "therapeutics"[All Fields] OR "therapies"[All Fields] OR "therapy"[Subheading] OR "therapy"[All Fields] OR "therapy's"[All Fields] OR "therapys"[All Fields]  Apexogenesis: "apexification"[MeSH Terms] OR "apexification"[All Fields] OR "apexogenesis"[All Fields]  Laser: "laser's"[All Fields] OR "lasers"[MeSH Terms] OR "lasers"[All Fields] OR "laser"[All Fields] OR "lasered"[All Fields] OR "lasering"[All Fields]  Mineral Trioxide Aggregate: "mineral trioxide aggregate"[Supplementary Concept] OR "mineral trioxide aggregate"[All Fields]  Calcium Hydroxide: "calcium hydroxide"[MeSH Terms] OR ("calcium"[All Fields] AND "hydroxide"[All Fields]) OR "calcium hydroxide"[All Fields]  Biodentine: "tricalcium silicate"[Supplementary Concept] OR "tricalcium silicate"[All Fields] OR "biodentine"[All Fields]  Formocresol: "formocresol"[Supplementary Concept] OR "formocresol"[All Fields]  Electrosurgical,: "electrosurgical"[All Fields] OR "electrosurgically"[All Fields]  Calcium: "calcium"[MeSH Terms] OR "calcium"[All Fields] OR "calciums"[All Fields] OR "calcium's"[All Fields]  Enriched: "enrich"[All Fields] OR "enriched"[All Fields] OR "enriches"[All Fields] OR "enriching"[All Fields] OR "enrichment"[All Fields] OR "enrichments"[All Fields]  Mixture: "mixture"[All Fields] OR "mixture's"[All Fields] OR "mixtures"[All Fields]  Simvastatin: "simvastatin"[MeSH Terms] OR "simvastatin"[All Fields] OR "simvastatin's"[All Fields] OR "simvastatins"[All Fields]  Zinc Oxide Eugenol: "zinc oxide-eugenol cement"[MeSH Terms] OR ("zinc"[All Fields] AND "oxide-eugenol"[All Fields] AND "cement"[All Fields]) OR "zinc oxide-eugenol cement"[All Fields] OR ("zinc"[All Fields] AND "oxide"[All Fields] AND "eugenol"[All Fields]) OR "zinc oxide eugenol"[All Fields]  Sodium Hypochlorite: "sodium hypochlorite"[MeSH Terms] OR ("sodium"[All Fields] AND "hypochlorite"[All Fields]) OR "sodium hypochlorite"[All Fields]  Primary Tooth: "tooth, deciduous"[MeSH Terms] OR ("tooth"[All Fields] AND "deciduous"[All Fields]) OR "deciduous tooth"[All Fields] OR ("primary"[All Fields] AND "tooth"[All Fields]) OR "primary tooth"[All Fields]  Milk Tooth: "tooth, deciduous"[MeSH Terms] OR ("tooth"[All Fields] AND "deciduous"[All Fields]) OR "deciduous tooth"[All Fields] OR ("milk"[All Fields] AND "tooth"[All Fields]) OR "milk tooth"[All Fields]  Deciduous Tooth: "tooth, deciduous"[MeSH Terms] OR ("tooth"[All Fields] AND "deciduous"[All Fields]) OR "deciduous tooth"[All Fields] OR ("deciduous"[All Fields] AND "tooth"[All Fields])  Temporary: "temporaries"[All Fields] OR "temporary"[All Fields]  tooth: "teeth's"[All Fields] OR "teeths"[All Fields] OR "tooth"[MeSH Terms] OR "tooth"[All Fields] OR "teeth"[All Fields] OR "tooth's"[All Fields] OR "tooths"[All Fields]  Systematic review: "systematic review"[Publication Type] .or. "systematic reviews as topic"[MeSH Terms] .or. "systematic review"[All Fields]  Review: "review"[Publication Type] .or. "review literature as topic"[MeSH Terms] .or. "review"[All Fields]  Meta analysis: "meta-analysis"[Publication Type] .or. "meta-analysis as topic"[MeSH Terms] .or. "meta-analysis"[All Fields] |
| LILACS | 46 | (pulpotomy OR pulp therapy OR vital pulp therapy OR pt OR apexogenesis) AND (primary tooth OR milk tooth OR deciduous tooth OR temporary tooth ) AND (systematic review OR review OR meta analysis) |
| SCOPUS | 29 | ( TITLE-ABS-KEY ( pulpotomy  OR  pulp  AND therapy  OR  vital  AND pulp  AND therapy  OR  pet  OR  amelogenesis )  AND  TITLE-ABS-KEY ( primary  AND tooth  OR  milk  AND tooth  OR  deciduous  AND tooth  OR  temporary  AND tooth )  AND  TITLE-ABS-KEY ( systematic  AND review  OR  review  OR  meta  AND analysis ) ) |
| EMBASE | 22 | (pulpotomy:ti,ab,kw OR 'pulp therapy':ti,ab,kw OR 'vital pulp therapy':ti,ab,kw OR pt:ti,ab,kw OR apexogenesis:ti,ab,kw OR laser:ti,ab,kw OR mta:ti,ab,kw OR 'mineral trioxide aggregate':ti,ab,kw OR 'calcium hydroxide':ti,ab,kw OR biodentine:ti,ab,kw OR formocresol:ti,ab,kw OR electrosurgical:ti,ab,kw OR 'calcium enriched mixture':ti,ab,kw OR simvastatin:ti,ab,kw OR 'zinc oxide eugenol':ti,ab,kw OR 'sodium hypochlorite':ti,ab,kw) AND ('primary tooth':ti,ab,kw OR 'milk tooth':ti,ab,kw OR 'deciduous tooth':ti,ab,kw OR 'temporary tooth':ti,ab,kw) AND ('systematic review':ti,ab,kw OR review:ti,ab,kw OR 'meta analysis':ti,ab,kw) |
| Web of Science | 11 | (Pulpotomy OR Pulp therapy OR Vital Pulp Therapy OR PT OR Apexogenesis) AND TITLE: (Primary Tooth OR Milk Tooth OR Deciduous Tooth OR Temporary tooth) AND TITLE: (Systematic Review OR Review OR Meta analysis) |
| COCHRANE | 07 | Pulpotomy OR Pulp therapy OR Vital Pulp Therapy OR PT OR Apexogenesis in Title Abstract Keyword AND Primary Tooth OR Milk Tooth OR Deciduous Tooth OR Temporary tooth in Title Abstract Keyword - (Word variations have been searched) |
| Total | 452 |  |

| Classification | Criteria |
| --- | --- |
| Convincing Evidence (Class I) | 1. More than 100 cases 2. Significant summary associations (p<1x10^-6^) 3. No evidence of small-study effects 4. No large heterogeneity (I^2^≤50%) 5. Low risk of bias in included primary studies (>50% primary studies showing low Risk of Bias with none having high Risk of Bias) |
| Highly Suggestive Evidence (Class II) | 1. More than 100 cases 2. Significant summary associations (p<1x10^-6^) 3. No large heterogeneity (I^2^≤50%) 4. Low risk of bias in included primary studies (>50% primary studies showing low Risk of Bias with none having high Risk of Bias) |
| Suggestive Evidence (Class III) | 1. More than 100 cases 2. Significant summary associations (p<1x10^-3^) 3. No large heterogeneity (I^2^≤50%) 4. Low risk of bias in included primary studies (>50% primary studies showing low Risk of Bias with none having high Risk of Bias) |
| Weak Evidence (Class IV) | 1. All summary associations with p<0.05 2. Significant Heterogeneity (I^2^>50%) 3. Moderate/High risk of bias in included primary studies (>50% primary studies showing Moderate/Unclear Risk of Bias with at least one having high Risk of Bias) |
| Negligible Evidence (Class V) | 1. All summary associations with p>0.05 2. Significant Heterogeneity (I^2^>50%) 3. Moderate/High risk of bias in included primary studies (>50% primary studies showing Moderate/Unclear Risk of Bias with at least one having high Risk of Bias) |

**Appendix 2: Proposed criteria for classification of the evidence of the outcomes of the meta-analysis of intervention studies in dental research**

Appendix 3: List of the excluded studies with reasons for exclusion

**Primary studies:**

1. Chakraborty A, Dey B, Jana S. A Nonconventional Approach to Formocresol Pulpotomy. Int J Clin Pediatr Dent, 2018;11(6):490-495
2. Hui-Derksen EK, Chen CF, Majewski R, Tootla RG, Boynton JR. Retrospective record review: reinforced zinc oxide-eugenol pulpotomy: a retrospective study. Pediatr Dent. 2013 Jan-Feb;35(1):43-6.
3. Borba de Araújo F, Eduardo Nor J, Thomazi TH. Formocresol diluído. Uma alternativa para a terapia pulpar em dentes decíduos [Dilute formocresol. An alternative in pulp therapy for deciduous teeth]. RGO. 1988 May-Jun;36(3):181-4.
4. Smith NL, Seale NS, Nunn ME. Ferric sulfate pulpotomy in primary molars: a retrospective study. Pediatr Dent. 2000 May-Jun;22(3):192-9

**Narrative review:**

1. Papagiannoulis L. Clinical studies on ferric sulphate as a pulpotomy medicament in primary teeth. Eur J Paediatr Dent. 2002 Sep;3(3):126-32.
2. Cohenca N, Paranjpe A, Berg J. Vital pulp therapy. Dent Clin North Am. 2013 Jan;57(1):59-73.
3. Frankl SN. Pulp therapy in pedodontics. Oral Surg Oral Med Oral Pathol. 1972 Aug;34(2):293-309.
4. Fuks AB, Papagiannoulis L. Pulpotomy in primary teeth: review of the literature according to standardized criteria. Eur Arch Paediatr Dent. 2006 Jun;7(2):64-71
5. Amrollahi P, Shah B, Seifi A, Tayebi L. Recent advancements in regenerative dentistry: A review, *Materials Science& Engineering C* (2016), doi: 10.1016/j.msec.2016.08.045
6. Al-Dlaigan YH. Pulpotomy Medicaments used in Deciduous Dentition: An Update. J Contemp Dent Pract. 2015 Jun 1;16(6):486-503.
7. AAPD. Use of Vital Pulp Therapies in Primary Teeth with Deep Caries Lesions. Pediatr Dent. 2017 Sep 15;39(6):173-186.
8. Brosnan MG, Natarajan AK, Campbell JM, Drummond BK. Management of the pulp in primary teeth--an update. N Z Dent J. 2014 Dec;110(4):119-23.
9. Darío Cárdenas J. Terapia pulpar en dentición decidua. Formocresol vs. glutaraldehido, una revision de literatura [Pulp therapy in the deciduous dentition. Formocresol vs. glutaraldehyde, a review of the literature]. Acta Clin Odontol. 1987 Jun;10(19):11-5
10. Frankl SN. Pulp therapy in pedodontics. Oral Surg Oral Med Oral Pathol. 1972 Aug;34(2):293-309.
11. Fuks AB. Pulpotomy in primary teeth. Eur Arch Paediatr Dent. 2006 Sep;7(3):124.
12. Fuks AB, Papagiannoulis L. Pulpotomy in primary teeth: review of the literature according to standardized criteria. Eur Arch Paediatr Dent. 2006 Jun;7(2):64-71
13. Fuks AB, Eidelman E. Pulp therapy in the primary dentition. Curr Opin Dent. 1991 Oct;1(5):556-63.
14. Mamaladze M, Ustiashvili M. Clinical parallels in treatment of reversible pulpitis. Georgian Med News. 2013 Jun;(219):14-22.
15. Ng FK, Messer LB. Mineral trioxide aggregate as a pulpotomy medicament: a narrative review. Eur Arch Paediatr Dent. 2008 Mar;9(1):4-11.
16. Papagiannoulis L. Clinical studies on ferric sulphate as a pulpotomy medicament in primary teeth. Eur J Paediatr Dent. 2002 Sep;3(3):126-32.
17. Pilipili, C. M. and Vanden Abbeele, A. and van den Abbeele, K. Pulpotomy of deciduous teeth. Rev Belge Med Dent. 2004;59:156-62.
18. Ranly DM. Pulp therapy in primary teeth. A review and prospectus. Acta Odontol Pediatr. 1982 Dec;3(2):63-8.
19. Ranly D M, Garcia-Godoy F. Current and potential pulp therapies for primary and young permanent teeth. J Dent. 2000; 28:153-61.
20. Razi RS. Pulp therapy in the primary dentition. N Y State Dent J. 1999 Mar;65(3):18-22.
21. Ripa LW. Review. Pulp therapy for the primary dentition. I. The treatment of teeth with vital pulps. J Conn State Dent Assoc. 1970 Jul;44(3):193-9.
22. Ritwik P. A review of pulp therapy for primary and immature permanent teeth. J Calif Dent Assoc. 2013 Aug;41(8):585-95.
23. Stringhini Junior E, Oliveira LB, Abanto J, Moura ACVM, Navarro RS, Imparato JCP. Evidências científicas atuais sobre a terapia pulpar de dentes decíduos. Rev. Assoc. Paul. Cir. Dent. 2014;68:259-262.
24. Udin RD. The formocresol pulpotomy revisited: looking at alternatives. J Calif Dent Assoc. 1991 Sep;19(9):27-34.
25. Wong BJ, Fu E, Mathu-Muju KR. Thirty-Month Outcomes of Biodentine ® Pulpotomies in Primary Molars: A Retrospective Review. Pediatr Dent. 2020 Jul 15;42(4):293-299.

**Risk of bias not evaluated/ Clear:**

1. Ansari G, Safi Aghdam H, Taheri P, Ghazizadeh Ahsaie M. Laser pulpotomy-an effective alternative to conventional techniques-a systematic review of literature and meta-analysis. Lasers Med Sci. 2018 Nov;33(8):1621-1629.
2. de Alencar Filho AV, Dos Santos Junior VE, da Silva Calixto M, Santos N, Heimer MV, Rosenblatt A. Evaluation of the genotoxic effects of formocresol application in vital pulp therapy of primary teeth: a clinical study and meta-analysis. Clin Oral Investig. 2018 Sep;22(7):2553-2558.
3. Peng L, Ye L, Tan H, Zhou X. Evaluation of the formocresol versus mineral trioxide aggregate primary molar pulpotomy: a meta-analysis. Oral Surg Oral Med Oral Pathol Oral Radiol Endod. 2006 Dec;102(6):e40-4.
4. Lin PY, Chen HS, Wang YH, Tu YK. Primary molar pulpotomy: a systematic review and network meta-analysis. J Dent. 2014 Sep;42(9):1060-77.
5. Asgary S, Shirvani A, Fazlyab M. MTA and ferric sulfate in pulpotomy outcomes of primary molars: a systematic review and meta-analysis. J Clin Pediatr Dent. 2014 Fall;39(1):1-8.
6. Loh A, O'Hoy P, Tran X, Charles R, Hughes A, Kubo K, Messer LB. Evidence-based assessment: evaluation of the formocresol versus ferric sulfate primary molar pulpotomy. Pediatr Dent. 2004 Sep-Oct;26(5):401-9.
7. Natalino LN, Paula FA, Teixeira MNC, Thiemy SV, da Silveira MAB, Moreira MMADA et al. Pulp therapy on primary teeth: evidence-based therapeutic possibilities. Rev. odontol. UNESP. 2013; 42 (2): 130-137
8. Subramanyam D, Somasundaram S. Clinical and radiographic outcome of herbal medicine versus standard pulpotomy medicaments in primary molars: A systematic review. Journal of Clinical and Diagnostic Research. 2017; 11: ZE12-ZE16.
9. Stringhini Junior E, Vitcel ME, Oliveira LB. Evidence of pulpotomy in primary teeth comparing MTA, calcium hydroxide, ferric sulphate, and electrosurgery with formocresol. Eur Arch Paediatr Dent. 2015 Aug;16(4):303-12.

**Unrelated:**

1. Guelmann M, Shapira J, Silva DR, Fuks AB. Esthetic restorative options for pulpotomized primary molars: a review of literature. J Clin Pediatr Dent. 2011 Winter;36(2):123-6.
2. Aïem E, Joseph C, Garcia A, Smaïl-Faugeron V, Muller-Bolla M. Caries removal strategies for deep carious lesions in primary teeth: Systematic review. Int J Paediatr Dent. 2020 Jul;30(4):392-404.
3. Benetti F, Lemos CAA, de Oliveira Gallinari M, Terayama AM, Briso ALF, de Castilho Jacinto R, Sivieri-Araújo G, Cintra LTA. Influence of different types of light on the response of the pulp tissue in dental bleaching: a systematic review. Clin Oral Investig. 2018 May;22(4):1825-1837.
4. Coll JA, Dhar V, Vargas K, Chen CY, Crystal YO, AlShamali S, Marghalani AA. Use of Non-Vital Pulp Therapies in Primary Teeth. Pediatr Dent. 2020 Sep 15;42(5):337-349.
5. Coll JA, Vargas K, Marghalani AA, Chen CY, AlShamali S, Dhar V, Crystal YO. A Systematic Review and Meta-Analysis of Nonvital Pulp Therapy for Primary Teeth. Pediatr Dent. 2020 Jul 15;42(4):256-461.
6. Gadallah L, Hamdy M, El Bardissy A, Abou El Yazeed M. Pulpotomy versus pulpectomy in the treatment of vital pulp exposure in primary incisors. A systematic review and meta-analysis. F1000Res. 2018 Sep 26;7:1560
7. Tedesco TK, Reis TM, Mello-Moura ACV, Silva GSD, Scarpini S, Floriano I, Gimenez T, Mendes FM, Raggio DP. Management of deep caries lesions with or without pulp involvement in primary teeth: a systematic review and network meta-analysis. Braz Oral Res. 2020 Nov 13;35:e004.
8. Smaïl-Faugeron V, Porot A, Muller-Bolla M, Courson F. Indirect pulp capping versus pulpotomy for treating deep carious lesions approaching the pulp in primary teeth: a systematic review. Eur J Paediatr Dent. 2016 Jun;17(2):107-12.
9. Ranly DM, Garcia-Godoy F. Current and potential pulp therapies for primary and young permanent teeth. J Dent. 2000 Mar;28(3):153-61.
10. Camp JH. Overviews of pediatric-endodontics. Alpha Omegan. 1991 Fall;84(2):26-7.
11. Cronshaw M, Parker S, Anagnostaki E, Mylona V, Lynch E, Grootveld M. Photobiomodulation Dose Parameters in Dentistry: A Systematic Review and Meta-Analysis. Dent J (Basel). 2020 Oct 6;8(4):114.
12. de Alencar Filho AV, Dos Santos Junior VE, da Silva Calixto M, Santos N, Heimer MV, Rosenblatt A. Evaluation of the genotoxic effects of formocresol application in vital pulp therapy of primary teeth: a clinical study and meta-analysis. Clin Oral Investig. 2018 Sep;22(7):2553-2558.

**One database searched-**

1. Anthonappa RP, King NM, Martens LC. Is there sufficient evidence to support the long-term efficacy of mineral trioxide aggregate (MTA) for endodontic therapy in primary teeth? Int Endod J. 2013 Mar;46(3):198-204.
2. Bossù M, Iaculli F, Di Giorgio G, Salucci A, Polimeni A, Di Carlo S. Different Pulp Dressing Materials for the Pulpotomy of Primary Teeth: A Systematic Review of the Literature. J Clin Med. 2020 Mar 19;9(3):838.
3. Gopalakrishnan V, Anthonappa R, Ekambaram M, King NM. Qualitative assessment of published studies on pulpotomy medicaments for primary molar teeth. J Investig Clin Dent. 2019 May;10(2):e12389.

**Updated Version included-**

1. Smaïl-Faugeron V, Courson F, Durieux P, Muller-Bolla M, Glenny AM, Fron Chabouis H. Pulp treatment for extensive decay in primary teeth. Cochrane Database Syst Rev. 2014 Aug 6;(8):CD003220.

**Appendix 4 : The overall quality of the included systematic reviews using AMSTAR-2**

| **Question/study** | **De Coster et al** | **Marghalani et al** | **Coll JA et al** | **Nematollahi et al** | **Nuvvula et al** | **Smaïl-Faugeron et al** | **Stringhini Junior et al** | **Jayaraman et al** |
| --- | --- | --- | --- | --- | --- | --- | --- | --- |
| **Did the research questions and inclusion criteria for the review include the components of PICO?** | Yes | Yes | Yes | Yes | Yes | Yes | Yes | Yes |
| **Did the report of the review contain an explicit statement that the review methods were established prior to the conduct of the review and did the report justify any significant deviations from the protocol?** | No | No | Yes | No | Yes | Yes | No | Yes |
| **Did the review authors explain their selection of the study designs for inclusion in the review?** | Yes | Yes | Yes | Yes | Yes | Yes | Yes | Yes |
| **Did the review authors use a comprehensive literature search strategy?** | Yes | Yes | Yes | Yes | Yes | Yes | Yes | Yes |
| **Did the review authors perform study selection in duplicate?** | Yes | Yes | Yes | Yes | Yes | Yes | Yes | Yes |
| **Did the review authors perform data extraction in duplicate?** | Yes | Yes | Yes | Yes | Yes | Yes | Yes | Yes |
| **Did the review authors provide a list of excluded studies and justify the exclusions?** | No | No | Yes | No | Yes | Yes | No | Yes |
| **Did the review authors describe the included studies in adequate detail?** | Yes | Yes | Yes | Yes | Yes | Yes | Yes | Yes |
| **Did the review authors use a satisfactory technique for assessing the risk of bias (RoB) in individual studies that were included in the review?** | Yes | Yes | Yes | Yes | Yes | Yes | Yes | Yes |
| **Did the review authors report on the sources of funding for the studies included in the review?** | No | Yes | Yes | No | No | Yes | No | No |
| **If meta-analysis was performed did the review authors use appropriate methods for statistical combination of results?** | No meta-analysis conducted | Yes | Yes | Yes | No meta-analysis conducted | Yes | Yes | Yes |
| **If meta-analysis was performed, did the review authors assess the potential impact of RoB in individual studies on the results of the meta-analysis or other evidence synthesis?** | No meta-analysis conducted | Yes | Yes | Yes | No meta-analysis conducted | Yes | Yes | Yes |
| **Did the review authors account for RoB in individual studies when interpreting/ discussing the results of the review?** | Yes | Yes | Yes | Yes | Yes | Yes | Yes | Yes |
| **Did the review authors provide a satisfactory explanation for, and discussion of, any heterogeneity observed in the results of the review?** | Yes | Yes | Yes | Yes | No | Yes | Yes | Yes |
| **If they performed quantitative synthesis did the review authors carry out an adequate investigation of publication bias (small study bias) and discuss its likely impact on the results of the review?** | No meta-analysis conducted | No | NA | No | No meta-analysis conducted | No | No | NA |
| **Did the review authors report any potential sources of conflict of interest, including any funding they received for conducting the review?** | Yes | Yes | Yes | Yes | Yes | Yes | Yes | Yes |
| **Overall Confidence** | Critically Low | Critically Low | High | Critically Low | High | Low | Critically low | High |

**Appendix 5: Details of the reporting characteristics of the included systematic reviews**

|  | Checklist/ Author | De Coster et al | Marghalani et al | Coll JA et al | Nematollahi et al | Nuvvula et al | Smaïl-Faugeron et al | Stringhini Junior et al | Jayaraman et al |
| --- | --- | --- | --- | --- | --- | --- | --- | --- | --- |
| Title 1) | Identify the report as a systematic review in title. | Yes | Yes | Yes | Yes | Yes | Yes | Yes | Yes |
| Abstract 2) | Report an abstract addressing each item in the PRISMA 2020 for Abstracts checklist. | Yes | Yes | Yes | Yes | Yes | Yes | Yes | Yes |
| Introduction | Rationale 3): Describe the current state of knowledge and its uncertainties. | Yes | Yes | Yes | Yes | Yes | Yes | Yes | Yes |
|  | Objectives 4): Provide an explicit statement of all objective(s) or question(s) the review addresses, expressed in terms of a relevant question formulation framework | Yes | Yes | Yes | Yes | Yes | Yes | Yes | Yes |
|  | Eligibility criteria 5):Specify the inclusion and exclusion criteria for the review and how studies were grouped for the syntheses. | Yes | No | Yes | Yes | Yes | Yes | Yes | Yes |
|  | Information sources 6): Specify all databases, registers, websites, organisations, reference lists and other sources searched or consulted to identify studies. Specify the date when each source was last searched or consulted. | Yes | Yes | Yes | Yes | Yes | Yes | Yes | Yes |
|  | Search strategy 7): Present the full search strategies for all databases, registers and websites, including any filters and limits used. | Yes | Yes | Yes | No | Yes | Yes | No | Yes |
|  | Selection process 8): Specify the methods used to decide whether a study met the inclusion criteria of the review, including how many reviewers screened each record and each report retrieved, whether they worked independently, and if applicable, details of automation tools used in the process | Yes | Yes | Yes | Yes | Yes | Yes | Yes | Yes |
|  | Data collection process 9): Specify the methods used to collect data from reports, including how many reviewers collected data from each report, whether they worked independently, any processes for obtaining or confirming data from study investigators, and if applicable, details of automation tools used in the process | Yes | Yes | Yes | Yes | Yes | Yes | Yes | Yes |
|  | Data items 10a): List and define all outcomes for which data were sought. Specify whether all results that were compatible with each outcome domain in each study were sought (e.g. for all measures, time points, analyses), and if not, the methods used to decide which results to collect | Yes | Yes | Yes | Yes | Yes | Yes | Yes | Yes |
|  | Data items 10 b): List and define all other variables for which data were sought (e.g. participant and intervention characteristics, funding sources). Describe any assumptions made about any missing or unclear information. | Yes | Yes | Yes | Yes | Yes | Yes | Yes | Yes |
|  | Study risk of bias assessment 11): Specify the methods used to assess risk of bias in the included studies, including details of the tool(s) used, how many reviewers assessed each study and whether they worked independently, and if applicable, details of automation tools used in the process | Yes | Yes | Yes | Yes | Yes | Yes | Yes | Yes |
|  | Effect measures 12): Specify for each outcome the effect measure(s) (e.g. risk ratio, mean difference) used in the synthesis or presentation of results. | NA | Yes | Yes | Yes | NA | Yes | Yes | Yes |
|  | Synthesis methods 13a): Describe the processes used to decide which studies were eligible for each synthesis (e.g. tabulating the study intervention characteristics and comparing against the planned groups for each synthesis (item #5)). | Yes | Yes | Yes | Yes | Yes | Yes | Yes | Yes |
|  | Synthesis methods 13b) Describe any methods required to prepare the data for presentation or synthesis, such as handling of missing summary statistics, or data conversions. | Yes | Yes | Yes | Yes | Yes | Yes | No | Yes |
|  | Synthesis methods 13c) Describe any methods used to tabulate or visually display results of individual studies and syntheses. | Yes | Yes | Yes | Yes | Yes | Yes | Yes | Yes |
|  | Synthesis methods 13d) Describe any methods used to synthesize results and provide a rationale for the choice(s). If meta-analysis was performed, describe the model(s), method(s) to identify the presence and extent of statistical heterogeneity, and software package(s) used. | NA | Yes | Yes | Yes | NA | Yes | Yes | Yes |
|  | Synthesis methods 13e) Describe any methods used to explore possible causes of heterogeneity among study results (e.g. subgroup analysis, meta-regression). | NA | Yes | Yes | Yes | NA | Yes | No | No |
|  | Synthesis methods 13f): Describe any sensitivity analyses conducted to assess robustness of the synthesized results. | NA | Yes | Yes | Yes | NA | Yes | No | No |
|  | Reporting bias assessment 14): Describe any methods used to assess risk of bias due to missing results in a synthesis (arising from reporting biases). | NA | No | NA | No | NA | No | No | NA |
|  | Certainty assessment 15): Describe any methods used to assess certainty (or confidence) in the body of evidence for an outcome. | NA | No | Yes | No | NA | No | Yes | Yes |
| RESULTS | Study selection 16a) Describe the results of the search and selection process, from the number of records identified in the search to the number of studies included in the review, ideally using a flow diagram | Yes | Yes | Yes | Yes | Yes | Yes | Yes | Yes |
|  | Study selection 16b) : Cite studies that might appear to meet the inclusion criteria, but which were excluded, and explain why they were excluded | Yes | Yes | Yes | Yes | Yes | Yes | No | Yes |
|  | Study characteristics 17): Cite each included study and present its characteristics. | Yes | Yes | Yes | Yes | Yes | Yes | Yes | Yes |
|  | Risk of bias in studies 18): Present assessments of risk of bias for each included study. | Yes | Yes | Yes | Yes | Yes | Yes | Yes | Yes |
|  | Results of individual studies 19): For all outcomes, present, for each study: (a) summary statistics for each group (where appropriate) and (b) an effect estimate and its precision (e.g. confidence/credible interval), ideally using structured tables or plots. | NA | Yes | Yes | Yes | NA | Yes | Yes | Yes |
|  | Results of syntheses 20a): For each synthesis, briefly summarise the characteristics and risk of bias among contributing studies. | Yes | Yes | Yes | Yes | Yes | Yes | Yes | Yes |
|  | Results of syntheses 20b): Present results of all statistical syntheses conducted. If meta-analysis was done, present for each the summary estimate and its precision (e.g. confidence/credible interval) and measures of statistical heterogeneity. If comparing groups, describe the direction of the effect. | NA | Yes | Yes | Yes | NA | Yes | Yes | Yes |
|  | Results of syntheses 20c): Present results of all investigations of possible causes of heterogeneity among study results | NA | Yes | Yes | Yes | NA | Yes | Yes | Yes |
|  | Results of syntheses 20d): Present results of all sensitivity analyses conducted to assess the robustness of the synthesized results | NA | No | Yes | No | NA | No | No | No |
|  | Reporting biases 21): Present assessments of risk of bias due to missing results (arising from reporting biases) for each synthesis assessed | NA | No | NA | No | NA | No | No | NA |
|  | Certainty of evidence 22): Present assessments of certainty (or confidence) in the body of evidence for each outcome assessed. | NA | No | Yes | No | NA | No | Yes | Yes |
| DISCUSSION | 23a) Provide a general interpretation of the results in the context of other evidence. | Yes | Yes | Yes | Yes | Yes | Yes | Yes | Yes |
|  | 23b)Discuss any limitations of the evidence included in the review.. | Yes | Yes | Yes | Yes | Yes | Yes | Yes | Yes |
|  | 23c) Discuss any limitations of the review processes used. | No | Yes | Yes | Yes | Yes | Yes | Yes | Yes |
|  | 23d) Discuss implications of the results for practice, policy, and future research. | Yes | Yes | Yes | Yes | Yes | Yes | Yes | Yes |
| Registration and protocol | 24 a)Provide registration information for the review, including register name and registration number, or state that the review was not registered. | No | No | Yes | No | Yes | Yes | No | Yes |
|  | 24b)Indicate where the review protocol can be accessed, or state that a protocol was not prepared. | No | No | Yes | No | Yes | Yes | No | Yes |
|  | 24c)Describe and explain any amendments to information provided at registration or in the protocol. | NA | NA | Yes | NA | NA | NA | No | Yes |
| Support | 25 )Describe sources of financial or non-financial support for the review, and the role of the funders or sponsors in the review. | Yes | Yes | No | Yes | Yes | Yes | No | No |
| Competing interests | 26) Declare any competing interests of review authors. | No | No | Yes | Yes | Yes | Yes | Yes | Yes |
| Availability of data, code and other materials | 27) Report which of the following are publicly available and where they can be found: template data collection forms; data extracted from included studies; data used for all analyses; analytic code; any other materials used in the review | No | No | Yes | Yes | No | Yes | No | Yes |
|  | Yes | 24 | 31 | 39 | 33 | 28 | 36 | 29 | 36 |
|  | Total | 29 | 41 | 40 | 41 | 29 | 41 | 42 | 40 |
|  | percentage | 82.8 | 75.6 | 97.5 | 75.6 | 96.55 | 87.8 | 69.04 | 90 |

**Appendix 6: Details of the primary studies included in the systematic reviews along-with their overlap (Red colour showing overlap in systematic reviews, yellow showing overlap in meta-analysis)**

| SNo | Author | Year | De Coster et al | Marghalani et al | Coll et al | Nematollahi et al | Nuvvula et al | | Smaïl-Faugeron et al | Stringhini Junior et al | Jayaraman et al |
| --- | --- | --- | --- | --- | --- | --- | --- | --- | --- | --- | --- |
| 1. 1 | Alaçam et al | 1989 |  |  |  |  |  | | 1 |  |  |
| 1. 2 | Fei et al | 1991 |  |  | 1 |  | 1 | | 2 |  | 1 |
| 1. 3 | Fishman et al | 1996 |  |  |  |  |  | | 3 |  |  |
| 1. 4 | Fuks et al | 1997 |  |  | 2 |  | 2 | | 4 |  |  |
| 1. 5 | Elliott RD et al | 1999 |  |  |  | 1 |  | |  |  |  |
| 1. 6 | Shumayrikh NM and Adenubi JO | 1999 |  |  | 3 |  |  | | 5 |  |  |
| 1. 7 | Ibricevic H and Al-Jame G | 2000 |  |  | 4 |  |  | | 6 |  |  |
| 1. 8 | Nadkarni U and Damle SG | 2000 |  |  |  |  |  | | 7 |  |  |
| 1. 9 | Waterhouse PJ et al | 2000 |  |  | 5 |  |  | | 8 |  |  |
| 1. 10 | Eidelman E et al | 2001 |  |  | 6 |  |  | | 9 |  |  |
| 1. 11 | Dean JA et al | 2002 |  |  |  |  |  | | 10 |  |  |
|  | Falster CA et al | 2002 |  |  | 7 |  |  | |  |  |  |
| 1. 12 | Pescheck A et al | 2002 | 1 |  |  |  |  | |  |  |  |
|  | Casas MJ et al | 2003 |  |  | 8 |  |  | |  |  |  |
| 1. 13 | Ibricevic H and Al-Jame Q | 2003 |  |  | 9 |  | 3 | |  |  |  |
| 1. 14 | Agamy HA et al | 2004 |  |  | 10 |  |  | | 11 |  |  |
| 1. 15 | Casas MJ et al | 2004 |  |  | 11 |  |  | | 12 |  |  |
|  | Jabbarifar SE et al | 2004 |  |  | 12 |  |  | |  |  |  |
| 1. 16 | Mortazavi M and Mesbahi M | 2004 |  |  |  |  |  | | 13 |  |  |
| 1. 17 | Farsi N et al | 2005 |  | 1 | 13 |  |  | | 14 |  |  |
| 1. 18 | Holan G et al | 2005 |  |  | 14 |  |  | | 15 |  |  |
| 1. 19 | Huth KC et al | 2005 |  |  | 15 |  |  | | 16 |  |  |
| 1. 20 | Markovic D et al | 2005 |  |  | 16 |  | 4 | | 17 |  | 2 |
| 1. 21 | Naik S and Hegde AH | 2005 |  |  |  |  |  | | 18 |  |  |
| 1. 22 | Ozalp N et al | 2005 |  |  |  |  |  | | 19 |  |  |
| 1. 23 | Saltzman B et al | 2005 | 2 |  | 17 | 2 |  | | 20 |  |  |
| 1. 24 | Furze HA and Furze ME | 2006 | 3 |  |  |  |  | |  |  |  |
| 1. 25 | Liu JF | 2006 | 4 |  |  | 3 |  | |  |  |  |
|  | Marchi JJ et al | 2006 |  |  | 18 |  |  | |  |  |  |
| 1. 26 | Neamatollahi H and Tajik A | 2006 |  |  |  |  | 5 | |  |  |  |
| 1. 27 | Vargas KG et al | 2006 |  |  | 19 |  | 6 | | 21 |  |  |
| 1. 28 | Aeinehchi M et al | 2007 |  |  |  |  |  | | 22 |  |  |
| 1. 29 | Demir T and Cehreli ZC | 2007 |  |  | 20 |  |  | | 23 |  |  |
| 1. 30 | Odabas ME et al | 2007 | 5 |  |  | 4 | 7 | |  |  |  |
|  | Aminabadi NA et al | 2008 |  |  | 21 |  |  | |  |  |  |
| 1. 31 | Bahrololoomi Z et al | 2008 |  |  |  |  |  | | 24 |  |  |
|  | Buyukgural B and Cehreli ZC | 2008 |  |  | 22 |  |  | |  |  |  |
|  | Casagrande L et al | 2008 |  |  | 23 |  |  | |  |  |  |
| 1. 32 | Coser RM et al | 2008 |  |  |  |  |  | | 25 |  |  |
| 1. 33 | Moretti AB et al | 2008 |  |  | 24 |  |  | | 26 |  |  |
| 1. 34 | Noorollahian H | 2008 |  | 2 | 25 |  |  | | 27 |  |  |
| 1. 35 | Prabhakar AR et al | 2008 |  |  |  |  |  | | 28 |  |  |
|  | Roberts JD | 2008 |  |  | 26 |  |  | |  |  |  |
| 1. 36 | Sabbarini J et al | 2008 |  |  |  |  |  | | 29 |  |  |
| 1. 37 | Sonmez D et al | 2008 |  |  | 27 |  | 8 | | 30 |  | 3 |
| 1. 38 | Trairatvorakul C and Chunlasikaiwan S | 2008 |  |  |  |  |  | | 31 |  |  |
| 1. 39 | Tuna D and Olmez A | 2008 |  |  | 28 |  |  | | 32 |  |  |
| 1. 40 | Zurn D and Seale NS | 2008 |  |  | 29 |  |  | | 33 |  |  |
| 1. 41 | Alaçam et al | 2009 |  |  | 30 |  |  | | 34 |  |  |
|  | Casagrande et al | 2009 |  |  | 31 |  |  | |  |  |  |
| 1. 42 | Garrocho-Rangel A et al | 2009 |  |  |  |  |  | | 35 |  |  |
| 1. 43 | Golpayegani MV et al | 2009 |  |  |  | 5 |  | |  |  |  |
| 1. 44 | Haghgoo R et al | 2009 |  |  |  |  |  | | 36 |  |  |
| 1. 45 | Sakai VT et al | 2009 |  |  | 32 |  |  | | 37 |  |  |
| 1. 46 | Subramaniam P et al | 2009 |  | 3 | 33 |  |  | | 38 |  |  |
| 1. 47 | Aminabadi NA et al | 2010 |  |  | 34 |  |  | | 39 |  |  |
| 1. 48 | Ansari G and Ranjpour M | 2010 |  |  |  |  |  | | 40 |  |  |
|  | Casagrande et al | 2010 |  |  | 35 |  |  | |  |  |  |
| 1. 49 | Doyle TL et al | 2010 |  |  | 36 |  | 9 | | 41 |  |  |
|  | Golpayegani MV et al | 2010 | 6 |  |  |  |  | |  |  |  |
| 1. 50 | Nakornchai S et al | 2010 |  |  |  |  |  | | 42 |  |  |
| 1. 51 | Ramar K and Mungara J | 2010 |  |  |  |  |  | | 43 |  |  |
| 1. 52 | Zealand CM et al | 2010 |  |  |  |  |  | | 44 |  |  |
| 1. 53 | Erdem AP et al | 2011 |  |  | 37 |  | 10 | | 45 |  | 4 |
| 1. 54 | Gisoure EF | 2011 |  |  |  |  | 11 | |  |  |  |
| 1. 55 | Liu H et al | 2011 |  |  | 38 |  |  | | 46 |  |  |
| 1. 56 | Malekafzali B et al | 2011 |  |  | 39 |  |  | | 47 |  |  |
|  | Nematollahi H et al | 2011 |  |  | 40 |  |  | |  |  |  |
|  | Odabas ME et al | 2011 |  |  | 41 |  |  | |  |  |  |
| 1. 57 | Pinky C eta l | 2011 |  |  |  |  |  | | 48 |  |  |
|  | Srinivasan D and Jayanthi M | 2011 |  |  | 42 |  |  | |  |  |  |
| 1. 58 | Subramaniam P and Gilhotra K | 2011 |  |  |  |  |  | | 49 |  |  |
|  | Howley B et al | 2012 |  |  | 43 |  |  | |  |  |  |
| 1. 59 | Huth KC et al | 2012 | 7 |  | 44 | 6 | 12 | |  |  | 5 |
|  | Odabas ME et al | 2012 |  |  | 45 |  |  | |  |  |  |
| 1. 60 | Sushynski JM et al | 2012 |  | 4 | 46 |  |  | |  |  |  |
|  | Trairatvorakul C and Detsomboonrat P | 2012 |  |  | 47 |  |  | |  |  |  |
|  | Yaman E et al | 2012 |  |  | 48 |  |  | |  |  |  |
| 1. 61 | Celik B et al | 2013 |  |  | 49 |  |  | | 50 |  |  |
| 1. 62 | Fernández CC et al | 2013 |  | 5 | 50 |  | 13 | | 51 |  | 6 |
| 1. 63 | Fallahinejad Ghajari M et al | 2013 |  |  | 51 |  |  | | 52 |  |  |
| 1. 64 | Havale R et al | 2013 |  |  | 52 |  | 14 | |  |  |  |
| 1. 65 | Oliveira TM et al | 2013 |  |  |  |  |  | | 53 |  |  |
|  | Ruby JD et al | 2013 |  |  | 53 |  |  | |  |  |  |
| 1. 66 | Shabzendedar M et al | 2013 |  |  | 54 |  |  | | 54 |  |  |
|  | Fouad WA and Yossef R | 2013 |  |  |  |  |  | |  | 1 |  |
|  | Al-Mutairi MA and Bawazir OA | 2013 |  |  | 55 |  |  | |  |  |  |
| 1. 67 | Akcay M and Sari S | 2014 |  |  | 56 |  |  | | 55 |  |  |
| 1. 68 | Cantekin K and Gümü H | 2014 |  |  | 57 |  |  | | 56 |  |  |
| 1. 69 | Chandra SP et al | 2014 |  |  |  |  |  | | 57 |  |  |
| 1. 70 | Durmus B and Tanboga I | 2014 |  |  | 58 | 7 | 15 | | 58 |  |  |
| 1. 71 | Goyal S et al | 2014 |  |  |  |  |  | | 59 |  |  |
| 1. 72 | Jayam C et al | 2014 |  |  | 59 |  |  | | 60 |  |  |
| 1. 73 | Khorakian F et al | 2014 |  |  | 60 |  |  | | 61 |  |  |
| 1. 74 | Rewal N et al | 2014 |  |  |  |  |  | | 62 |  |  |
|  | Trairatvorakul C and Sastararuji T | 2014 |  |  | 61 |  |  | |  |  |  |
| 1. 75 | Ulusoy AT et al | 2014 |  |  | 62 |  |  | | 63 |  |  |
| 1. 76 | Yadav P et al | 2014 |  |  |  | 8 | 16 | | 64 |  |  |
| 1. 77 | Yildiz E and Tosun G | 2014 |  |  |  |  | 17 | |  |  |  |
| 1. 78 | Chen XX et al | 2015 |  |  |  |  |  | | 65 |  |  |
|  | Farsi DJ et al | 2015 |  |  | 63 |  |  | |  |  |  |
| 1. 79 | Fernandes AP et al | 2015 |  |  | 64 | 9 |  | | 66 |  |  |
| 1. 80 | Gupta G et al | 2015 |  |  |  | 10 | 18 | | 67 |  |  |
| 1. 81 | Kang CM et al | 2015 |  |  |  |  |  | | 68 |  |  |
| 1. 82 | Kusum B et al | 2015 |  |  |  |  |  | | 69 | 2 |  |
| 1. 83 | Lourenço Neto N et al | 2015 |  |  |  |  |  | | 70 |  |  |
| 1. 84 | Niranjani K et al | 2015 |  |  |  | 11 |  | | 71 | 3 |  |
| 1. 85 | Olatosi OO et al | 2015 |  |  | 65 |  |  | | 72 |  |  |
| 1. 86 | Al-Ostwani AO et al | 2016 |  |  |  |  |  | | 73 |  |  |
| 1. 87 | Aminabadi NA et al | 2016 |  |  | 66 |  |  | | 74 |  |  |
| 1. 88 | Arikan V et al | 2016 |  |  |  |  |  | | 75 |  |  |
| 1. 89 | Bezgin T et al | 2016 |  |  |  |  |  | | 76 |  |  |
| 1. 90 | Cuadros-Fernández C et al | 2016 |  |  | 67 |  |  | | 77 | 4 |  |
| 1. 91 | El Meligy OA et al | 2016 |  |  |  |  |  | | 78 |  |  |
| 1. 92 | Goyal P et al | 2016 |  |  |  |  | 19 | | 79 |  |  |
| 1. 93 | Grewal N et al | 2016 |  |  |  |  |  | | 80 |  |  |
|  | Musale PK and Soni AS | 2016 |  |  | 68 |  |  | |  |  |  |
| 1. 94 | Pramila R et al | 2016 |  |  |  |  |  | | 81 |  |  |
|  | Togaru H et al | 2016 |  |  |  |  |  | | 82 | 5 |  |
| 1. 96 | Yildirim C et al | 2016 |  |  | 69 |  |  | | 83 |  |  |
|  | Uloopi KS et al | 2016 |  |  | 70 | 12 |  | | 84 |  |  |
| 1. 97 | Kalra M et al | 2017 |  |  |  |  |  | | 85 |  |  |
| 1. 98 | Nguyen TD et al | 2017 |  |  |  |  |  | | 86 |  |  |
| 1. 99 | Ozmen B and Bayrak S | 2017 |  |  |  |  |  | | 87 |  | 7 |
| 1. 100 | Rajasekharan S et al | 2017 |  |  | 71 |  |  | | 88 | 6 |  |
| 1. 102 | Bani M et al | 2017 |  |  |  |  |  | |  | 7 |  |
| 1. 103 | Carti O and Oznurhan F | 2017 |  |  |  |  |  | |  | 8 |  |
| 1. 104 | Juneja P and Kulkarni S | 2017 |  |  |  |  |  | |  | 9 |  |
| 1. 106 | Ansari G et al | 2018 |  |  |  |  |  | |  |  | 8 |
|  |  |  | 7/0 | 5/0 | 71/11 | 12/11 | 19/0 | | 88/36 | 9/9 | 8/8 |
|  | Overlap measure | Overall overlap of the primary studies | | | | | | Overlap of primary studies included in meta-analysis | | | |
| A | % Overlap of primary studies (Studies common to at least two reviews/ Total number of studies)x100 | (59/129)x100=45.73%  **Moderate overlap** | | | | | | 19/52=36.54%  **Moderate Overlap** | | | |
| B | Covered Area (CA)  {(Total number of included publications in each review- inclusive of double counting)/ (Total number of studies-rows)x(Total number of columns)}x100 | {(7+5+71+12+19+88+9+8)/(129x8)}x100=  {219/1032}x100=21.22%  **Moderate Overlap** | | | | | | {(11+11+36+9+8)/(52x5)}x100=  {75/260}x100=28.85%  **High Overlap** | | | |
| C | Corrected Covered Ares (CCA)  {(Total number of included publications in each review- inclusive of double counting)-Total number of studies/ (Total number of studies-rows)x(Total number of columns)-Total number of studies}x100 | {(7+5+71+12+19+88+9+8)-129/(129x8)-129}x100= {90/903}x100=9.97%  **Moderate Overlap** | | | | | | {(11+11+36+9+8)-52/(52x5)-52}x100=  {23/208}x100=11.06%  **High Overlap** | | | |

**Appendix 7: Details of the included meta-analyses for different comparisons along with the small study effect and level of evidence**

| **Author** | **Quality of SR** | **Comparisons** | **Studies** | **ROB in studies** | **Year** | **Sample Size A** | **Sample Size B** | **RR** | **OR** | **RD** | **I2** | **P** | **Small Study effect** | **Overall Confidence** |  |
| --- | --- | --- | --- | --- | --- | --- | --- | --- | --- | --- | --- | --- | --- | --- | --- |
| **MTA Vs Full strength/ 1:5 Dilution Formocresol** | | | | | | | | | | | | | | | |
| Smail-Faugeron et al 2018 | Low | Clinical-6 months | 13 | High-8, Unclear-5 ,Low-0 | 2004-16 | 517/518 | 526/530 | 1.01 [0.99, 1.02] | 2.48 [0.40, 15.21] | 0.01 [-0.01, 0.02] | I² = 0% | 0.45 | Absent | NE |  |
|  | Low | Clinical-12 months | 12 | High-6,Unclear-6, Low-0 | 2004-16 | 380/383 | 347/357 | 1.01 [0.99, 1.03] | 2.99 [0.90, 9.92] | 0.01 [-0.01, 0.03] | I² = 0% | 0.48 | Absent | NE |  |
|  | Low | Clinical-24 months | 9 | High-5, Unclear-4, Low-0 | 2005-16 | 270/275 | 262/273 | 1.01 [0.99, 1.03] | 2.17 [0.68, 6.96] | 0.01 [-0.01, 0.03] | I² = 0% | 0.46 | Absent | NE |  |
|  | Low | Radiographic- 6 months | 12 | High-7, Unclear-5, Low-0 | 2004-16 | 460/467 | 435/455 | 1.01 [0.99, 1.03] | 2.63 [1.08, 6.37] | 0.01 [-0.01, 0.03] | I² = 0% | 0.36 | Absent | NE |  |
|  | Low | Radiographic- 12 months | 12 | High-6, Unclear-6 ,Low-0 | 2004-16 | 375/383 | 339/357 | 1.01 [0.99, 1.04] | 2.33 [0.94, 5.78] | 0.01 [-0.01, 0.04] | I² = 16% | 0.32 | Absent | NE |  |
|  | Low | Radiographic- 24 months | 9 | High-5, Unclear-4, Low-0 | 2005-16 | 264/275 | 246/273 | 1.05 [1.00, 1.10] | 2.30 [0.97, 5.44] | 0.05 [0.00, 0.09] | I² = 22% | 0.05 | Absent | Weak Evidence- Class IV |  |
|  | Low | Overall-6 months | 6 | High-4, Unclear-2, Low-0 | 2008-14 | 164/165 | 157/163 | 1.02 [0.98, 1.05] | 3.99 [0.60, 26.69] | 0.02 [-0.02, 0.05] | I² = 0% | 0.41 | Absent | NE |  |
|  | Low | Overall-12 months | 6 | High-4,Unclear-2, Low-0 | 2008-14 | 161/165 | 154/163 | 1.01 [0.97, 1.06] | 1.95 [0.54, 6.99] | 0.02 [-0.02, 0.06] | I² = 4% | 0.48 | Absent | NE |  |
| Coll et al 2017 | High | Overall- 12 months | 11 | High-0, Unclear-11, Low-0 | 2001-16 | 339/361 | 288/326 | 1.03 [0.99, 1.07] |  | 0.03 (-0.01, 0.07) | I² = 15% | 0.2 | Absent | NE |  |
| Coll et al 2017 | High | Overall- 18 months | 4 | High-0, Unclear-4, Low-0 | 2008-13 | 118/128 | 111/131 | 1.04 [0.96, 1.11] |  | 0.04 (-0.03, 0.10) | I² = 19% | 0.33 | Absent | NE |  |
| Smail-Faugeron et al 2018 | Low | Overall-24 months | 7 | High-5, Unclear-2, Low-0 | 2008-14 | 175/185 | 163/183 | 1.05 [0.98, 1.12] | 1.98 [0.78, 5.04] | 0.05 [-0.02, 0.11] | I² = 34% | 0.2 | Absent | NE |  |
| Marghalani et al 2014 | Critically Low | Clinical-24 months | 5 | High-3, Unclear-2, Low-0 | 2005-13 | 156/156 | 159/161 | 1.01 [0.98, 1.05] | 3.12 [0.32, 30.57] | 0.01 [-0.02, 0.04] | I² = 0% | 0.41 | Absent | NE |  |
|  | Critically Low | Radiographic-24 months | 5 | High-3, Unclear-2, Low-0 | 2005-13 | 150/156 | 136/161 | 1.09 [0.97, 1.22] | 3.36 [1.06, 10.59] | 0.08 [-0.03, 0.18] | I² = 63% | 0.16 | Absent | NE |  |
| Coll et al 2017 | High | Overall- 24 months | 8 | High-0, Unclear-7, Low-1 | 2008-16 | 219/241 | 183/215 | 1.04 [0.98, 1.11] | 1.91 [1.04, 3.53] | 0.04 [-0.02, 0.10] | I² = 15% | 0.18 | Absent | NE |  |
| **MTA Vs Full strength Formocresol** | | | | | | | | | | | | | | | |
| Smail-Faugeron et al 2018 | Low | Clinical-6 months | 6 | High-5, Unclear-1, Low-0 | 2004-16 | 268/269 | 268/269 | 1.00 [0.98, 1.02] | 1.41 [0.14, 13.93] | 0.00 [-0.02, 0.02] | I² = 0% | 0.92 | Absent | NE |  |
|  | Low | Clinical-12 months | 5 | High-3, Unclear-2, Low-0 | 2004-16 | 225/228 | 199/204 | 1.01 [0.98, 1.03] | 2.14 [0.51, 8.94] | 0.01 [-0.02, 0.03] | I² = 0% | 0.6 | Absent | NE |  |
|  | Low | Clinical-24 months | 3 | High-2, Unclear-1, Low-0 | 2005-16 | 145/145 | 143/145 | 1.01 [0.98, 1.04] | 3.07 [0.31, 30.07] | 0.01 [-0.02, 0.04] | I² = 0% | 0.5 | Absent | NE |  |
|  | Low | Radiographic- 6 months | 4 | High-3, Unclear-1, Low-0 | 2004-16 | 192/193 | 165/169 | 1.01 [0.97, 1.04] | 2.73 [0.19, 40.20] | 0.01 [-0.03, 0.04] | I² = 35% | 0.63 | Absent | NE |  |
|  | Low | Radiographic- 12 months | 5 | High-3, Unclear-2, Low-0 | 2004-16 | 225/228 | 194/204 | 1.04 [0.98, 1.09] | 3.07 [0.85, 11.05] | 0.04 [-0.01, 0.08] | I² = 50% | 0.17 | Absent | NE |  |
|  | Low | Radiographic- 24 months | 3 | High-2, Unclear-1, Low-0 | 2005-16 | 143/145 | 131/145 | 1.09 [1.03, 1.15] | 4.80 [1.28, 17.96] | 0.08 [0.03, 0.13] | I² = 0% | 0.003 | Absent | Weak Evidence- Class IV |  |
|  | Low | Overall-6 months | 2 | High-1, Unclear-1, Low-0 | 2005-16 | 95/95 | 94/95 | 1.00 [0.97, 1.03] | 3.09 [0.12, 78.41] | 0.00 [-0.03, 0.03] | I² = 0% | 0.79 | Absent | NE |  |
|  | Low | Overall-12 months | 3 | High-1, Unclear-2, Low-0 | 2005-16 | 130/130 | 127/130 | 1.01 [0.98, 1.05] | 4.10 [0.44, 38.05] | 0.01 [-0.02, 0.05] | I² = 0% | 0.21 | Absent | NE |  |
|  | Low | Overall-24 months | 2 | High-1, Unclear-1, Low-0 | 2005-16 | 93/95 | 93/95 | 1.01 [0.97, 1.05] | 0.95 [0.14, 6.68] | 0.01 [-0.03, 0.05] | I² = 0% | 0.69 | Absent | NE |  |
| **MTA Vs 1:5 Dilution Formocresol** | | | | | | | | | | | | | | | |
| Smail-Faugeron et al 2018 | Low | Clinical-6 months | 8 | High-4, Unclear-4, Low-0 | 2008-15 | 274/274 | 283/286 | 1.01 [0.99, 1.03] | 6.41 [0.33, 125.39] | 0.01 [-0.01, 0.03] | I² = 0% | 0.29 | Absent | NE |  |
|  | Low | Clinical-12 months | 7 | High-3, Unclear-4, Low-0 | 2008-15 | 155/155 | 148/153 | 1.01 [0.97, 1.04] | 6.64 [0.73, 60.29] | 0.01 [-0.02, 0.05] | I² = 0% | 0.63 | Absent | NE |  |
|  | Low | Clinical-24 months | 6 | High-3, Unclear-3, Low-0 | 2008-13 | 125/130 | 119/128 | 1.01 [0.96, 1.06] | 2.25 [0.25, 19.99] | 0.01 [-0.04, 0.06] | I² = 15% | 0.73 | Absent | NE |  |
|  | Low | Radiographic- 6 months | 8 | High-4, Unclear-4, Low-0 | 2008-15 | 268/274 | 270/286 | 1.01 [0.98, 1.04] | 2.59 [0.98, 6.82] | 0.01 [-0.01, 0.04] | I² = 0% | 0.35 | Absent | NE |  |
|  | Low | Radiographic- 12 months | 7 | High-3, Unclear-4, Low-0 | 2008-15 | 150/155 | 144/153 | 1.00 [0.96, 1.05] | 1.90 [0.57, 6.30] | 0.01 [-0.03, 0.04] | I² = 0% | 0.84 | Absent | NE |  |
|  | Low | Radiographic- 24 months | 6 | High-3, Unclear-3, Low-0 | 2008-13 | 121/130 | 115/128 | 1.02 [0.95, 1.09] | 1.46 [0.48, 4.48] | 0.02 [-0.05, 0.09] | I² = 27% | 0.65 | Absent | NE |  |
|  | Low | Overall-6 months | 4 | High-1, Unclear-3, Low-0 | 2005-15 | 100/100 | 100/100 | 1.00 [0.96, 1.04] | Not estimable | 0.00 [-0.04, 0.04] | I² = 0% | 1 | Absent | NE |  |
|  | Low | Overall-12 months | 3 | High-1, Unclear-2, Low-0 | 2011-15 | 75/75 | 75/75 | 1.00 [0.96, 1.04] | Not estimable | 0.00 [-0.04, 0.04] | I² = 0% | 1 | Absent | NE |  |
|  | Low | Overall-24 months | 2 | High-1, Unclear-1, Low-0 | 2011-13 | 49/50 | 50/50 | 0.99 [0.93, 1.05] | 0.32 [0.01, 8.25] | -0.01 [-0.07, 0.05] | I² = 0% | 0.68 | Absent | NE |  |
| **MTA Vs Calcium Hydroxide** | | | | | | | | | | | | | | | |
| Smail-Faugeron et al 2018 | Low | Clinical-6 months | 4 | High-2, Unclear-2, Low-0 | 2008-14 | 76/76 | 72/74 | 1.01 [0.96, 1.06] | 5.74 [0.25, 130.37] | 0.01 [-0.04, 0.06] | I² = 0% | 0.8 | Absent | NE |  |
|  | Low | Clinical-12 months | 4 | High-2, Unclear-2, Low-0 | 2008-14 | 76/76 | 64/74 | 1.14 [0.95, 1.35] | 6.46 [1.34, 31.24] | 0.14 [-0.02, 0.29] | I² = 66% | 0.15 | Absent | NE |  |
|  | Low | Clinical-24 months | 5 | High-2, Unclear-3, Low-0 | 2008-14 | 156/163 | 97/121 | 1.18 [0.99, 1.41] | 4.62 [1.73, 12.33] | 0.15 [0.01, 0.29] | I² = 71% | 0.07 | Absent | NE |  |
|  | Low | Radiographic- 6 months | 4 | High-2, Unclear-2, Low-0 | 2008-14 | 76/76 | 57/74 | 1.27 [0.96, 1.68] | 17.99 [3.24, 100.04] | 0.22 [0.01, 0.43] | I² = 82% | 0.09 | Absent | NE |  |
|  | Low | Radiographic- 12 months | 4 | High-2, Unclear-2, Low-0 | 2008-14 | 74/76 | 48/74 | 1.44 [1.20, 1.71] | 9.67 [2.71, 34.45] | 0.33 [0.19, 0.48] | I² = 8% | < 0.0001 | Absent | Weak Evidence- Class IV |  |
|  | Low | Radiographic- 24 months | 5 | High-2, Unclear-3, Low-0 | 2008-14 | 153/163 | 64/121 | 1.73 [1.29, 2.31] | 12.94 [4.00, 41.82] | 0.41 [0.26, 0.57] | I² = 61% | 0.0002 | Absent | Weak Evidence- Class IV |  |
|  | Low | Overall-6 months | 2 | High-2, Unclear-0, Low-0 | 2008-11 | 35/35 | 31/33 | 1.04 [0.94, 1.16] | 5.54 [0.25, 123.08] | 0.04 [-0.06, 0.14] | I² = 0% | 0.47 | Absent | NE |  |
|  | Low | Overall-12 months | 2 | High-2, Unclear-0, Low-0 | 2008-11 | 32/35 | 25/33 | 1.20 [0.98, 1.48] | 3.54 [0.82, 15.25] | 0.16 [-0.01, 0.32] | I² = 0% | 0.08 | Absent | NE |  |
| Coll et al 2017 | High | Overall- 12 months | 5 | High-0, Unclear-5, Low-0 | 2008- 2014 | 158/ 164 | 75/ 122 | 1.50 [1.28, 1.76] |  |  | I² = 18% | 0.00001 | Absent | Suggestive Evidence Class III |  |
| Coll et al 2017 | High | Overall- 18 months | 3 | High-0, Unclear-5, Low-0 | 2008- 2012 | 109/ 116 | 39/ 74 | 1.63 [1,17, 2.28] |  |  | I² = 53% | 0.004 | Absent | Weak Evidence- Class IV |  |
| Smail-Faugeron et al 2018 | Low | Overall-24 months | 2 | High-2, Unclear-0, Low-0 | 2008-11 | 29/35 | 20/33 | 1.37 [1.04, 1.81] | 3.48 [0.99, 12.30] | 0.24 [0.05, 0.43] | I² = 0% | 0.03 | Absent | Weak Evidence- Class IV |  |
| Coll et al 2017 | High | Overall- 24 months | 3 | High-0, Unclear-2, Low-1 | 2008-13 | 106/116 | 34/74 | 1.96 [1.52, 2.53] | 9.94 [1.96, 50.32] | 0.46 [0.30, 0.61] | I² = 0% | < 0.00001 | Absent | Suggestive Evidence Class III |  |
| **Biodentin Vs MTA** | | | | | | | | | | | | | | | |
| Smail-Faugeron et al 2018 | Low | Clinical-6 months | 4 | High-2, Unclear-2, Low-0 | 2015-17 | 111/115 | 117/119 | 0.99 [0.95, 1.04] | 0.61 [0.12, 3.19] | -0.01 [-0.05, 0.04] | I² = 0% | 0.76 | Absent | NE |  |
|  | Low | Clinical-12 months | 2 | High-1, Unclear-1, Low-0 | 2016-17 | 68/70 | 71/74 | 1.01 [0.92, 1.10] | 1.23 [0.12, 12.53] | 0.01 [-0.08, 0.09] | I² = 37% | 0.88 | Absent | NE |  |
|  | Low | Radiographic- 6 months | 4 | High-2, Unclear-2, Low-0 | 2015-17 | 109/115 | 117/119 | 0.98 [0.94, 1.03] | 0.41 [0.10, 1.74] | -0.02 [-0.06, 0.03] | I² = 10% | 0.46 | Absent | NE |  |
|  | Low | Radiographic- 12 months | 2 | High-1, Unclear-1, Low-0 | 2016-17 | 67/70 | 71/74 | 0.99 [0.93, 1.06] | 0.93 [0.16, 5.23] | -0.01 [-0.07, 0.05] | I² = 0% | 0.93 | Absent | NE |  |
| Stringhini Junior et al 2019 | Critically Low | Clinical-6 months | 9 | High-2, Unclear-6, Low-1 | 2013-17 | 241/245 | 246/248 | 1.00 [0.97, 1.02] | 0.61 [0.12, 3.18] | -0.00 [-0.03, 0.02] | I² = 0% | 0.87 | Absent | NE |  |
|  | Critically Low | Clinical-12 months | 6 | High-0, Unclear-5, Low-1 | 2016-17 | 175/178 | 177/181 | 1.01 [0.97, 1.05] | 1.20 [0.31, 4.69] | 0.01 [-0.02, 0.04] | I² = 0% | 0.61 | Absent | NE |  |
|  | Critically Low | Clinical-18 months | 3 | High-0, Unclear-2, Low-1 | 2017 | 67/69 | 70/71 | 0.99 [0.93, 1.05] | 0.59 [0.07, 4.96] | -0.01 [-0.07, 0.05] | I² = 0% | 0.7 | Absent | NE |  |
|  | Critically Low | Radiographic-6 months | 9 | High-2, Unclear-6, Low-1 | 2013-17 | 230/245 | 242/248 | 0.99 [0.96, 1.02] | 0.40 [0.16, 1.03] | -0.01 [-0.04, 0.02] | I² = 16% | 0.49 | Absent | NE |  |
|  | Critically Low | Radiographic-12 months | 6 | High-0, Unclear-5, Low-1 | 2016-17 | 163/178 | 171/181 | 1.00 [0.95, 1.05] | 0.61 [0.25, 1.48] | -0.01 [-0.05, 0.04] | I² = 18% | 0.9 | Absent | NE |  |
|  | Critically Low | Radiographic-18 months | 3 | High-0, Unclear-2, Low-1 | 2017 | 64/69 | 66/71 | 0.99 [0.90, 1.09] | 0.99 [0.28, 3.54] | -0.01 [-0.09, 0.08] | I² = 0% | 0.9 | Absent | NE |  |
| Coll et al 2017 | High | Overall-12 months | 2 | High-0, Unclear-2, Low-1 | 2016 |  |  | 1.01 [0.94,1.09] |  |  | I² = 0% | 0.83 | Absent | NE |  |
| **MTA Vs Ferric Sulphate** | | | | | | | | | | | | | | | |
| Smail-Faugeron et al 2018 | Low | Clinical-6 months | 4 | High-3, Unclear-1, Low-0 | 2008-16 | 95/95 | 89/95 | 1.03 [0.94, 1.13] | 16.18 [0.87, 301.62] | 0.04 [-0.05, 0.13] | I² = 67% | 0.48 | Absent | NE |  |
|  | Low | Clinical-12 months | 3 | High-2, Unclear-1, Low-0 | 2008-13 | 65/65 | 63/65 | 1.02 [0.96, 1.08] | 5.43 [0.25, 118.96] | 0.02 [-0.04, 0.07] | I² = 0% | 0.61 | Absent | NE |  |
|  | Low | Clinical-24 months | 3 | High-2, Unclear-1, Low-0 | 2008-13 | 60/65 | 55/65 | 1.09 [0.92, 1.29] | 2.21 [0.30, 16.53] | 0.08 [-0.07, 0.23] | I² = 41% | 0.31 | Absent | NE |  |
|  | Low | Radiographic- 6 months | 4 | High-3, Unclear-1, Low-0 | 2008-16 | 95/95 | 78/95 | 1.15 [0.89, 1.49] | 16.10 [0.72, 361.55] | 0.13 [-0.10, 0.37] | I² = 94% | 0.28 | Absent | NE |  |
|  | Low | Radiographic- 12 months | 3 | High-2, Unclear-1, Low-0 | 2008-13 | 63/65 | 62/65 | 1.01 [0.95, 1.08] | 1.34 [0.12, 14.86] | 0.01 [-0.05, 0.08] | I² = 0% | 0.7 | Absent | NE |  |
|  | Low | Radiographic- 24 months | 3 | High-2, Unclear-1, Low-0 | 2008-13 | 58/65 | 53/65 | 1.09 [0.95, 1.26] | 1.90 [0.45, 7.93] | 0.08 [-0.05, 0.21] | I² = 19% | 0.23 | Absent | NE |  |
|  | Low | Overall-6 months | 4 | High-3, Unclear-1, Low-0 | 2008-16 | 95/95 | 78/95 | 1.15 [0.89, 1.49] | 16.10 [0.72, 361.55] | 0.13 [-0.10, 0.37] | I² = 94% | 0.28 | Present | NE |  |
|  | Low | Overall-12 months | 3 | High-2, Unclear-1, Low-0 | 2008-13 | 63/65 | 62/65 | 0.93 [0.73, 1.18] | 0.46 [0.04, 5.75] | -0.07 [-0.28, 0.15] | I² = 0% | 0.7 | Absent | NE |  |
| Coll et al 2017 | High | Overall- 12 months | 4 | High-0, Unclear-4, Low-0 | 2008-13 | 92/ 102 | 64/103 | 1.17 [0.91, 1.49] |  |  | I² = 86% | 0.22 | Present | NE |  |
| Smail-Faugeron et al 2018 | Low | Overall-24 months | 3 | High-2, Unclear-1, Low-0 | 2008-13 | 58/65 | 56/65 | 1.05 [0.95, 1.17] | 1.32 [0.42, 4.17] | 0.05 [-0.05, 0.14] | I² = 0% | 0.64 | Absent | NE |  |
| Coll et al 2017 | High | Overall- 24 months | 4 | High-0, Unclear-3, Low-1 | 2008-13 | 99/107 | 80/100 | 1.13 [0.99, 1.29] | 2.78 [0.74, 10.45] | 0.11 [0.00, 0.22] | I² = 33% | 0.06 | Absent | NE |  |
| **MTA Vs Portland Cement** | | | | | | | | | | | | | | | |
| Smail-Faugeron et al 2018 | Low | Clinical-6 months | 3 | High-2, Unclear-1, Low-0 | 2009-16 | 65/65 | 63/65 | 1.03 [0.96, 1.10] | 5.30 [0.25, 114.47] | 0.03 [-0.04, 0.09] | I² = 0% | 0.43 | Absent | NE |  |
|  | Low | Clinical-12 months | 3 | High-2, Unclear-1, Low-0 | 2009-16 | 65/65 | 63/65 | 1.03 [0.96, 1.10] | 5.30 [0.25, 114.47] | 0.03 [-0.04, 0.09] | I² = 0% | 0.43 | Absent | NE |  |
|  | Low | Clinical-24 months | 3 | High-2, Unclear-1, Low-0 | 2009-16 | 65/65 | 63/65 | 1.03 [0.96, 1.10] | 5.30 [0.25, 114.47] | 0.03 [-0.04, 0.09] | I² = 0% | 0.43 | Absent | NE |  |
|  | Low | Radiographic- 6 months | 3 | High-2, Unclear-1, Low-0 | 2009-16 | 65/65 | 65/65 | 1.00 [0.95, 1.05] | Not estimable | 0.00 [-0.05, 0.05] | I² = 0% | 1 | Absent | NE |  |
|  | Low | Radiographic- 12 months | 3 | High-2, Unclear-1, Low-0 | 2009-16 | 65/65 | 65/65 | 1.00 [0.95, 1.05] | Not estimable | 0.00 [-0.05, 0.05] | I² = 0% | 1 | Absent | NE |  |
|  | Low | Radiographic- 24 months | 3 | High-2, Unclear-1, Low-0 | 2009-16 | 63/65 | 61/65 | 1.02 [0.94, 1.10] | 2.13 [0.36, 12.46] | 0.02 [-0.05, 0.09] | I² = 0% | 0.66 | Absent | NE |  |
| **MTA Vs Diode Laser** | | | | | | | | | | | | | | | |
| Nematolahi 2018 | Critically Low | Clinical-6-30 months | 1 | High-0, Unclear-0, Low- 1 | 2015 | * | * | * | 0.066 [0.00-11.29] | -0.123 | I² = 0% | 0.3 | Absent | NE |  |
|  | Critically Low | Radiographic-6-30 months | 2 | High- 0, Moderate-1, Low-1 | 2015-16 | * | * | * | 0.183 [0.023-1.478] | -0.134 | I² = 0% | 0.111 | Absent | NE |  |
| **Calcium Hydroxide Vs Formocresol** | | | | | | | | | | | | | | | |
| Smail-Faugeron et al 2018 | Low | Clinical-6 months | 6 | High-1, Unclear-5, Low-0 | 2000-15 | 130/158 | 158/174 | 0.93 [0.83, 1.04] | 0.28 [0.06, 1.25] | -0.07 [-0.17, 0.02] | I² = 69% | 0.2 | Absent | NE |  |
|  | Low | Clinical-12 months | 6 | High-1, Unclear-5, Low-0 | 2000-15 | 118/158 | 150/174 | 0.90 [0.73, 1.09] | 0.32 [0.05, 1.87] | -0.11 [-0.28, 0.06] | I² = 87% | 0.28 | Absent | NE |  |
|  | Low | Clinical-24 months | 3 | High-1, Unclear-2, Low-0 | 2005-08 | 63/72 | 74/78 | 0.94 [0.86, 1.02] | 0.41 [0.12, 1.47] | -0.06 [-0.14, 0.01] | I² = 0% | 0.13 | Absent | NE |  |
|  | Low | Radiographic- 6 months | 4 | High-1, Unclear-3, Low-0 | 2008-15 | 47/76 | 77/78 | 0.66 [0.37, 1.19] | 0.04 [0.01, 0.16] | -0.32 [-0.63, -0.00] | I² = 93% | 0.17 | Absent | NE |  |
|  | Low | Radiographic- 12 months | 6 | High-1, Unclear-5, Low-0 | 2000-15 | 86/158 | 130/174 | 0.70 [0.49, 1.00] | 0.20 [0.05, 0.78] | -0.26 [-0.46, -0.05] | I² = 82% | 0.05 | Absent | Weak Evidence- Class IV |  |
|  | Low | Radiographic- 24 months | 3 | High-1, Unclear-2, Low-0 | 2005-08 | 63/72 | 74/78 | 0.94 [0.86, 1.02] | 0.41 [0.12, 1.47] | -0.06 [-0.14, 0.01] | I² = 0% | 0.13 | Absent | NE |  |
|  | Low | Overall-12 months | 2 | High-1, Unclear-1, Low-0 | 2005-08 | 48/57 | 59/63 | 0.92 [0.82, 1.03] | 0.36 [0.10, 1.28] | -0.08 [-0.18, 0.02] | I² = 0% | 0.13 | Absent | NE |  |
| Coll et al 2017 | High | Overall- 12 months | 7 | High-0, Unclear-6, Low-1 | 2000-2014 | 160/ 174 | 102/ 162 | 1.43 [1.14, 1.79] |  |  | I² = 67% | 0.002 | Absent | Weak Evidence- Class IV |  |
| Coll et al 2017 | High | Overall- 18 months | 5 | High-0, Unclear-4, Low-1 | 2000-2014 | 123/ 136 | 84/ 116 | 1.25 [1.02, 1.53] |  |  | I² = 52% | 0.03 | Absent | Weak Evidence- Class IV |  |
| Smail-Faugeron et al 2018 | Low | Overall-24 months | 2 | High-1, Unclear-1, Low-0 | 2005-08 | 38/57 | 56/63 | 0.77 [0.64, 0.93] | 0.24 [0.09, 0.65] | -0.21 [-0.35, -0.07] | I² = 0% | 0.008 | Absent | Weak Evidence- Class IV |  |
| Coll et al 2017 | High | Overall- 24 months | 4 | High-0, Unclear-4, Low-0 | 2008- 2012 | 87/111 | 42/ 101 | 1.76 [1.40, 2.23] |  |  | I² = 0% | 0.00001 | Absent | Suggestive Evidence Class III |  |
| **Calcium Hydroxide Vs Ferric Sulphate** | | | | | | | | | | | | | | | |
| Smail-Faugeron et al 2018 | Low | Clinical-6 months | 2 | High-1, Unclear-1, Low-0 | 2005-08 | 56/57 | 65/65 | 0.98 [0.93, 1.04] | 0.29 [0.01, 7.23] | -0.02 [-0.07, 0.03] | I² = 0% | 0.48 | Absent | NE |  |
|  | Low | Clinical-12 months | 2 | High-1, Unclear-1, Low-0 | 2005-08 | 55/57 | 65/65 | 0.97 [0.92, 1.03] | 0.28 [0.03, 2.78] | -0.03 [-0.08, 0.03] | I² = 0% | 0.28 | Absent | NE |  |
|  | Low | Clinical-24 months | 2 | High-1, Unclear-1, Low-0 | 2005-08 | 50/57 | 63/65 | 0.93 [0.85, 1.01] | 0.25 [0.05, 1.26] | -0.08 [-0.16, 0.00] | I² = 0% | 0.08 | Absent | NE |  |
|  | Low | Radiographic- 12 months | 2 | High-1, Unclear-1, Low-0 | 2005-08 | 48/57 | 57/65 | 0.92 [0.67, 1.26] | 0.58 [0.08, 4.18] | -0.08 [-0.34, 0.18] | I² = 60% | 0.59 | Absent | NE |  |
|  | Low | Radiographic- 24 months | 2 | High-1, Unclear-1, Low-0 | 2005-08 | 38/57 | 54/65 | 0.82 [0.67, 1.01] | 0.39 [0.16, 0.94] | -0.16 [-0.31, -0.01] | I² = 0% | 0.06 | Absent | NE |  |
|  | Low | Overall-12 months | 2 | High-1, Unclear-1, Low-0 | 2005-08 | 48/57 | 57/65 | 0.92 [0.67, 1.26] | 0.58 [0.08, 4.18] | -0.08 [-0.34, 0.18] | I² = 60% | 0.59 | Absent | NE |  |
| Coll et al 2017 | High | Overall- 12 months | 2 | High-0, Unclear-2, Low-0 | 2008- 2012 | 57/ 65 | 46/ 56 | 1.10 [0.84, 1.45] |  |  | I² = 49% | 0.49 | Absent | NE |  |
| Coll et al 2017 | High | Overall- 18 months | 2 | High-0, Unclear-2, Low-0 | 2008- 2012 | 56/ 65 | 43/ 53 | 1.04 [0.89, 1.22] |  |  | I² = 0% | 0.59 | Absent | NE |  |
| Smail-Faugeron et al 2018 | Low | Overall-24 months | 2 | High-1, Unclear-1, Low-0 | 2005-08 | 38/57 | 54/65 | 0.82 [0.67, 1.01] | 0.39 [0.16, 0.94] | -0.16 [-0.31, -0.01] | I² = 0% | 0.06 | Absent | NE |  |
| Coll et al 2017 | High | Overall- 24 months | 2 | High-0, Unclear-2, Low-0 | 2008- 2012 | 54/65 | 28/53 | 1.57 [1.19, 2.06] |  |  | I² = 0% | 0.001 | Absent | Weak Evidence- Class IV |  |
| **Ferric Sulphate Vs Formocresol** | | | | | | | | | | | | | | | |
| Smail-Faugeron et al 2018 | Low | Clinical-6 months | 7 | High-3, Unclear-4, Low-0 | 1991-2017 | 198/199 | 194/195 | 1.00 [0.97, 1.02] | 1.00 [0.10, 10.02] | -0.00 [-0.03, 0.02] | I² = 0% | 0.87 | Absent | NE |  |
|  | Low | Clinical-12 months | 7 | High-3, Unclear-4, Low-0 | 1991-2017 | 194/199 | 192/195 | 0.99 [0.97, 1.02] | 0.78 [0.20, 3.12] | -0.01 [-0.03, 0.02] | I² = 0% | 0.74 | Absent | NE |  |
|  | Low | Clinical-24 months | 5 | High-2, Unclear-3, Low-0 | 2005-17 | 122/130 | 119/128 | 1.01 [0.96, 1.06] | 1.13 [0.39, 3.25] | 0.01 [-0.03, 0.05] | I² = 0% | 0.66 | Absent | NE |  |
|  | Low | Radiographic- 6 months | 6 | High-3, Unclear-3, Low-0 | 1991-2017 | 142/149 | 136/145 | 1.00 [0.95, 1.05] | 1.31 [0.34, 5.12] | 0.00 [-0.04, 0.05] | I² = 0% | 0.93 | Absent | NE |  |
|  | Low | Radiographic- 12 months | 7 | High-3, Unclear-4, Low-0 | 1991-2017 | 177/199 | 179/195 | 0.98 [0.91, 1.05] | 0.76 [0.29, 2.03] | 0.76 [0.29, 2.03] | I² = 30% | 0.51 | Absent | NE |  |
|  | Low | Radiographic- 24 months | 5 | High-2, Unclear-3, Low-0 | 2005-17 | 108/130 | 111/128 | 0.95 [0.86, 1.04] | 0.77 [0.38, 1.56] | -0.05 [-0.13, 0.03] | I² = 0% | 0.26 | Absent | NE |  |
|  | Low | Overall-6 months | 4 | High-2, Unclear-2, Low-0 | 1991-2013 | 92/94 | 86/90 | 1.00 [0.95, 1.06] | 1.62 [0.12, 22.22] | 0.00 [-0.05, 0.06] | I² = 5% | 0.91 | Absent | NE |  |
|  | Low | Overall-12 months | 5 | High-3, Unclear-2, Low-0 | 1991-2013 | 133/144 | 131/140 | 0.99 [0.91, 1.08] | 0.94 [0.17, 5.38] | -0.01 [-0.09, 0.07] | I² = 47% | 0.8 | Absent | NE |  |
| Coll et al 2017 | High | Overall- 12 months | 8 | High-0, Unclear-7, Low-1 | 1991- 2015 | 208/ 235 | 205/ 229 | 1.00 [0.93, 1.08] |  |  | I² = 50% | 0.99 | Absent | NE |  |
| Coll et al 2017 | High | Overall- 18 months | 5 | High-0, Unclear-4, Low-1 | 1997-2015 | 137/149 | 142/ 159 | 1.04 [ 0.95, 1.14] |  |  | I² = 29% | 0.4 | Absent | NE |  |
| Smail-Faugeron et al 2018 | Low | Overall-24 months | 4 | High-2, Unclear-2, Low-0 | 2005-13 | 98/115 | 102/113 | 0.94 [0.86, 1.03] | 0.64 [0.28, 1.46] | -0.05 [-0.13, 0.03] | I² = 0% | 0.21 | Absent | NE |  |
| Coll et al 2017 | High | Overall- 24 months | 4 | High-0, Unclear-4, Low-0 | 2008- 2013 | 99/ 112 | 89/ 104 | 1.02 [0.93, 1.13] |  |  | I² = 0% | 0.65 | Absent | NE |  |
| Jayaraman et al 2019 | High | Clinical-12 months | 6 | High-1, Unclear-5, Low-0 | 1991-2018 | 180/186 | 178/180 | 1.00 [0.98, 1.03] | 2.07 [0.41, 10.32] | 1.00 [0.98, 1.03] | I² = 0% | 0.77 | Absent | NE |  |
|  | High | Radiographic-12 months | 6 | High-1, Unclear-5, Low-0 | 1991-2018 | 172/184 | 171/180 | 0.98 [0.92, 1.04] | 0.80 [0.19, 3.45] | -0.02 [-0.07, 0.04] | I² = 33% | 0.51 | Absent | NE |  |
|  | High | Clinical-24 months | 5 | High-1, Unclear-4, Low-0 | 2008-17 | 118/130 | 117/128 | 0.98 [0.92, 1.06] | 0.90 [0.37, 2.18] | -0.01 [-0.08, 0.05] | I² = 0% | 0.65 | Absent | NE |  |
|  | High | Radiographic-24 months | 5 | High-1, Unclear-4, Low-0 | 2008-17 | 114/130 | 114/128 | 0.98 [0.91, 1.05] | 0.90 [0.41, 1.95] | -0.02 [-0.09, 0.05] | I² = 0% | 0.57 | Absent | NE |  |
| **Sodium Hypochlorite Vs Formocresol** | | | | | | | | | | | | | | | |
| Smail-Faugeron et al 2018 | Low | Clinical-6 months | 2 | High-2, Unclear-0, Low-0 | 2013 | 75/75 | 75/75 | 1.00 [0.97, 1.04] | Not estimable | 0.00 [-0.03, 0.03] | I² = 0% | 1 | Absent | NE |  |
|  | Low | Clinical-12 months | 2 | High-2, Unclear-0, Low-0 | 2013 | 75/75 | 75/75 | 1.00 [0.97, 1.04] | Not estimable | 0.00 [-0.03, 0.03] | I² = 0% | 1 | Absent | NE |  |
|  | Low | Radiographic- 6 months | 2 | High-2, Unclear-0, Low-0 | 2013 | 71/75 | 72/75 | 0.97 [0.82, 1.15] | 0.73 [0.03, 17.31] | -0.03 [-0.19, 0.13] | I² = 73% | 0.75 | Absent | NE |  |
|  | Low | Radiographic- 12 months | 2 | High-2, Unclear-0, Low-0 | 2013 | 69/75 | 72/75 | 0.95 [0.85, 1.08] | 0.52 [0.08, 3.57] | 0.95 [0.85, 1.08] | I² = 42% | 0.45 | Absent | NE |  |
| Coll et al 2017 | High | Overall- 12 months | 3 | High-0, Unclear-2, Low-1 | 2013- 2015 | 69/ 78 | 83/ 88 | 1.06 [0.97, 1.15] |  |  | I² = 0% | 0.22 | Absent | NE |  |
| Coll et al 2017 | High | Overall- 18 months | 2 | High-0, Unclear-1, Low-1 | 2013-15 | 34/41 | 50/50 | 0.83 [0.72, 0.96] | 0.09 [0.01, 0.71] | -0.17 [-0.29, -0.05] | I² = 0% | 0.01 | Absent | Weak Evidence- Class IV |  |
| **Ferric Sulphate Vs Diode Laser** | | | | | | | | | | | | | | | |
| Smail-Faugeron et al 2018 | Low | Clinical-6 months | 3 | High-0, Unclear-3, Low-0 | 2014-15 | 60/65 | 65/65 | 0.93 [0.81, 1.06] | 0.21 [0.03, 1.30] | -0.08 [-0.20, 0.04] | I² = 36% | 0.27 | Absent | NE |  |
|  | Low | Clinical-12 months | 2 | High-0, Unclear-2, Low-0 | 2014-15 | 46/50 | 50/50 | 0.94 [0.85, 1.04] | 0.18 [0.02, 1.59] | -0.07 [-0.19, 0.04] | I² = 5% | 0.21 | Absent | NE |  |
|  | Low | Radiographic- 6 months | 3 | High-0, Unclear-3, Low-0 | 2014-15 | 56/65 | 57/65 | 0.98 [0.86, 1.12] | 0.94 [0.33, 2.66] | 0.02 [-0.10, 0.15] | I² = 0% | 0.79 | Absent | NE |  |
|  | Low | Radiographic- 12 months | 2 | High-0, Unclear-2, Low-0 | 2014-15 | 39/50 | 40/50 | 0.94 [0.74, 1.19] | 0.78 [0.17, 3.64] | -0.06 [-0.29, 0.16] | I² = 25% | 0.63 | Absent | NE |  |
| Nematolahi et al 2018 | Critically Low | Clinical-6-30 months | 4 | High- 0, Moderate-2, Low-2 | 2012-15 | * | * | * | 2.346 [0.21-26.14] | 0.052 | I² = 0% | 0.488 | Absent | NE |  |
|  | Critically Low | Radiographic-6-30 months | 4 | High- 0, Moderate-2, Low-2 | 2012-15 | * | * | * | 0.873 [0.425-1.793] | 0.013 | I² = 0% | 0.711 | Absent | NE |  |
| Coll et al 2017 | High | Overall- 12 months | 2 | High-0, Unclear-2, Low-0 | 2012- 2014 | 74/ 87 | 74/ 90 | 1.06 [0.94, 1.19] |  |  | I² = 0% | 0.34 | Absent | NE |  |
| **Electrosurgery Vs Diode Laser** | | | | | | | | | | | | | | | |
| Smail-Faugeron et al 2018 | Low | Clinical-6 months | 2 | High-0, Unclear-2, Low-0 | 2014-15 | 23/25 | 25/25 | 0.93 [0.73, 1.20] | 0.16 [0.01, 3.85] | -0.07 [-0.30, 0.15] | I² = 53% | 0.6 | Absent | NE |  |
|  | Low | Radiographic- 6 months | 2 | High-0, Unclear-2, Low-0 | 2014-15 | 20/25 | 22/25 | 0.90 [0.70, 1.15] | 0.64 [0.14, 3.06] | -0.10 [-0.30, 0.10] | I² = 0% | 0.39 | Absent | NE |  |
| Nematolahi et al 2018 | Critically Low | Clinical-6-30 months | 2 | High- 0, Moderate-2, Low-0 | 2012-15 | * | * | * | 5.97 [0.05-717.28] | 0.054 | I² = 0% | 0.465 | Absent | NE |  |
|  | Critically Low | Radiographic-6-30 months | 2 | High- 0, Moderate-2, Low-0 | 2012-15 | * | * | * | 1.26 [0.22-7.06] | 0.106 | I² = 0% | 0.211 | Absent | NE |  |
| **Biodentin Vs Diode Laser** | | | | | | | | | | | | | | | |
| Nematolahi et al 2018 | Critically Low | Clinical-6-30 months | 1 | High- 0, Moderate-0, Low-1 | 2015 | * | * | * | 0.872 [0.107-7.092] | -0.015 | I² = 0% | 0.898 | Absent | NE |  |
|  | Critically Low | Radiographic-6-30 months | 1 | High- 0, Moderate-0, Low-1 | 2015 | * | * | * | 0.872 [0.107-7.092] | -0.015 | I² = 0% | 0.898 | Absent | NE |  |
| **Calcium Hydroxide Vs Diode Laser** | | | | | | | | | | | | | | | |
| Nematolahi et al 2018 | Critically Low | Clinical-6-30 months | 3 | High- 0, Moderate-0, Low-2 | 2012-15 | * | * | * | 2.474 [0.473-12.954] | 0.006 | I² = 0% | 0.283 | Absent | NE |  |
|  | Critically Low | Radiographic-6-30 months | 1 | High- 0, Moderate-0, Low-2 | 2012-15 | * | * | * | 1.8 [0.72-4.51] | 0.118 | I² = 0% | 0.211 | Absent | NE |  |
| Coll et al 2017 | High | Overall- 12 months | 2 | High-0, Unclear-1, Low-1 | 2012- 2014 | 56/ 62 | 45/ 57 | 1.11 [0.97, 1.27] |  |  | I² = 0% | 0.14 | Absent | NE |  |
| Coll et al 2017 | High | Overall- 18 months | 2 | High-0, Unclear-1, Low-1 | 2012- 2014 | 53/ 62 | 42/54 | 1.07 [0.91, 1.25] |  |  | I² = 0% | 0.41 | Absent | NE |  |
| **Formocresol Vs Diode Laser** | | | | | | | | | | | | | | | |
| Nematolahi et al 2018 | Critically Low | Clinical-6-30 months | 7 | High- 0, Moderate-2, Low-5 | 2005-15 | * | * | * | 1.03 [0.32-3.33] | 0.009 | I² = 0% | 0.963 | Absent | NE |  |
|  | Critically Low | Radiographic-6-30 months | 7 | High- 0, Moderate-2, Low-5 | 2012-15 | * | * | * | 0.55 [0.29-1.05] | 0.122 | I² = 0% | 0.071 | Absent | NE |  |
| Coll et al 2017 | High | Overall- 12 months | 3 | High-0, Unclear-2, Low-1 | 2012- 2014 | 98/ 105 | 86/ 102 | 1.08 [0.97, 1.20] |  |  | I² =20% | 0.15 | Absent | NE |  |
| Coll et al 2017 | High | Overall- 18 months | 2 | High-0, Unclear-1, Low-1 | 2012- 2014 | 61/64 | 53/ 62 | 1.14 [0.91, 1.43] |  |  | I² =51% | 0.27 | Absent | NE |  |
| **Sodium Hypochlorite Vs Ferric Sulphate** | | | | | | | | | | | | | | | |
| Smail-Faugeron et al 2018 | Low | Clinical-6 months | 2 | High-2, Unclear-0, Low-0 | 2006-13 | 57/57 | 53/53 | 1.00 [0.95, 1.05] | Not estimable | 0.00 [-0.05, 0.05] | I² = 0% | 1 | Absent | NE |  |
|  | Low | Clinical-12 months | 2 | High-2, Unclear-0, Low-0 | 2006-13 | 55/57 | 53/53 | 0.98 [0.92, 1.04] | 0.21 [0.01, 4.65] | -0.02 [-0.09, 0.04] | I² = 0% | 0.53 | Absent | NE |  |
|  | Low | Radiographic- 6 months | 2 | High-2, Unclear-0, Low-0 | 2006-13 | 51/57 | 43/53 | 1.09 [0.72, 1.66] | 1.39 [0.10, 19.53] | 0.07 [-0.26, 0.39] | I² = 85% | 0.68 | Absent | NE |  |
|  | Low | Radiographic- 12 months | 2 | High-2, Unclear-0, Low-0 | 2006-13 | 51/57 | 40/53 | 1.18 [0.72, 1.94] | 2.18 [0.23, 20.31] | 0.12 [-0.22, 0.47] | I² = 86% | 0.52 | Absent | NE |  |
| Coll et al 2017 | High | Overall- 12 months | 2 | High-0, Unclear-1, Low-1 | 2013- 2015 | 39/ 40 | 37/ 41 | 1.06 [0.95, 1.18] |  |  | I² = 0% | 0.28 | Absent | NE |  |
| Coll et al 2017 | High | Overall- 18 months | 2 | High-0, Unclear-1, Low-1 | 2013- 2015 | 36/ 40 | 37/ 40 | 0.99 [0.85, 1.16] |  |  | I² = 24% | 0.88 | Absent | NE |  |
| **Electro Surgery Vs Ferric Sulphate** | | | | | | | | | | | | | | | |
| Smail-Faugeron et al 2018 | Low | Clinical-6 months | 2 | High-0, Unclear-2, Low-0 | 2014-15 | 23/25 | 21/25 | 1.11 [0.91, 1.37] | 1.78 [0.30, 10.70] | 0.10 [-0.07, 0.27] | I² = 0% | 0.29 | Absent | NE |  |
|  | Low | Radiographic- 6 months | 2 | High-0, Unclear-2, Low-0 | 2014-15 | 20/25 | 21/25 | 0.95 [0.73, 1.23] | 0.76 [0.18, 3.28] | -0.04 [-0.25, 0.17] | I² = 0% | 0.69 | Absent | NE |  |
| **Ankaferd Blood Stopper Vs Ferric Sulphate** | | | | | | | | | | | | | | | |
| Smail-Faugeron et al 2018 | Low | Clinical-6 months | 2 | High-0, Unclear-2, Low-0 | 2014-17 | 50/50 | 50/50 | 1.00 [0.95, 1.05] | Not estimable | 0.00 [-0.05, 0.05] | I² = 0% | 1 | Absent | NE |  |
|  | Low | Clinical-12 months | 2 | High-0, Unclear-2, Low-0 | 2014-17 | 45/50 | 46/50 | 0.99 [0.89, 1.10] | 0.77 [0.19, 3.16] | -0.01 [-0.11, 0.08] | I² = 0% | 0.84 | Absent | NE |  |
|  | Low | Radiographic- 6 months | 2 | High-0, Unclear-2, Low-0 | 2014-17 | 48/50 | 48/50 | 1.00 [0.95, 1.06] | 1.00 [0.12, 8.21] | 0.00 [-0.05, 0.05] | I² = 0% | 1 | Absent | NE |  |
|  | Low | Radiographic- 12 months | 2 | High-0, Unclear-2, Low-0 | 2014-17 | 43/50 | 42/50 | 1.02 [0.87, 1.21] | 1.17 [0.39, 3.52] | 0.02 [-0.12, 0.16] | I² = 0% | 0.79 | Absent | NE |  |
| **Enamel matrix Derivative Vs Formocresol** | | | | | | | | | | | | | | | |
| Smail-Faugeron et al 2018 | Low | Clinical-6 months | 2 | High-0, Unclear-2, Low-0 | 2008-16 | 46/50 | 45/50 | 1.06 [0.76, 1.47] | 1.26 [0.08, 19.34] | 0.05 [-0.22, 0.32] | I² = 72% | 0.73 | Absent | NE |  |

**Appendix 8: Details of the pooled effect sizes of clinical, radiographic and overall success of different pulpotomy medicaments/techniques at different time periods (6 months, 12 months, 18 months, 24 months).**

| **Material** | **Criteria** | **Time period** | **No of studies** | **Year** | **Events-Pooled** | **Sample Size-Pooled** | **Success Rate Pooled** | **95% Confidence Interval** | **P Value** | **I2** |
| --- | --- | --- | --- | --- | --- | --- | --- | --- | --- | --- |
| MTA | Clinical | 6 months | 13 | 2008-2017 | 365 | 367 | 0.976 | 0.954,0.988 | 0.0001 | 0% |
| MTA | Clinical | 12 months | 13 | 2008-2017 | 367 | 373 | 0.968 | 0.943,0.983 | 0.0001 | 0% |
| MTA | Clinical | 18 months | 3 | 2017 | 70 | 71 | 0.972 | 0.895,0.993 | 0.0001 | 0% |
| MTA | Clinical | 24 months | 9 | 2008-2016 | 289 | 300 | 0.955 | 0.887,0.983 | 0.0001 | 57.51% |
| MTA | Radiographic | 6 months | 13 | 2008-2017 | 360 | 367 | 0.953 | 0.918,0.974 | 0.0001 | 4.58% |
| MTA | Radiographic | 12 months | 13 | 2008-2017 | 361 | 373 | 0.952 | 0.915,0.973 | 0.0001 | 17.74% |
| MTA | Radiographic | 24 months | 7 | 2008-2016 | 218 | 228 | 0.949 | 0.910,0.972 | 0.0001 | 0% |
| MTA | Overall | 6 months | 5 | 2005-2016 | 129 | 130 | 0.975 | 0.925,0.992 | 0.0001 | 0% |
| MTA | Overall | 12 months | 17 | 2001-2016 | 565 | 591 | 0.937 | 0.912,0.956 | 0.0001 | 0% |
| MTA | Overall | 18 months | 5 | 2008-2013 | 201 | 215 | 0.921 | 0.855,0.959 | 0.0001 | 26.66% |
| MTA | Overall | 24 months | 9 | 2008-2016 | 301 | 328 | 0.906 | 0.848,0.943 | 0.0001 | 36.16% |
| Formocresol Full Strength/Diluted | Clinical | 6 months | 12 | 2000-2017 | 314 | 336 | 0.951 | 0.875,0.981 | 0.0001 | 69.32% |
| Formocresol Full Strength/Diluted | Clinical | 12 months | 15 | 1991-2018 | 416 | 448 | 0.951 | 0.887,0.979 | 0.0001 | 74.28% |
| Formocresol Full Strength/Diluted | Clinical | 24 months | 8 | 2005-2016 | 221 | 235 | 0.931 | 0.855,0.969 | 0.0001 | 47.01% |
| Formocresol Full Strength/Diluted | Radiographic | 6 months | 9 | 2008-2017 | 217 | 225 | 0.941 | 0.894,0.968 | 0.0001 | 0% |
| Formocresol Full Strength/Diluted | Radiographic | 12 months | 15 | 1991-2017 | 392 | 458 | 0.906 | 0.796,0.960 | 0.0001 | 84.59% |
| Formocresol Full Strength/Diluted | Radiographic | 24 months | 7 | 2005-2017 | 162 | 181 | 0.873 | 0.782,0.929 | 0.0001 | 35.30% |
| Formocresol Full Strength/Diluted | Overall | 6 months | 2 | 2009-2011 | 43 | 45 | 0.933 | 0.761,0.983 | 0.0001 | 13.76% |
| Full strength Formocresol | Clinical | 6 months | 1 | 2016 | 34 | 35 | 0.971 | 0.823,0.996 | 0.001 | N/A |
| Full strength Formocresol | Clinical | 12 months | 2 | 2009-2016 | 66 | 70 | 0.955 | 0.869,0.985 | 0.0001 | 0% |
| Full strength Formocresol | Clinical | 24 months | 1 | 2016 | 34 | 35 | 0.971 | 0.823,0.996 | 0.001 | N/A |
| Full strength Formocresol | Radiographic | 6 months | 1 | 2016 | 35 | 35 | 0.986 | 0.813,0.999 | 0.003 | N/A |
| Full strength Formocresol | Radiographic | 12 months | 2 | 2009-2016 | 66 | 70 | 0.935 | 0.839,0.976 | 0.0001 | 0% |
| Full strength Formocresol | Radiographic | 24 months | 1 | 2016 | 30 | 35 | 0.857 | 0.699,0.939 | 0.0001 | N/A |
| Full strength Formocresol | Overall | 6 months | 1 | 2016 | 34 | 35 | 0.971 | 0.823,0.996 | 0.001 | N/A |
| Full strength Formocresol | Overall | 12 months | 2 | 2009-2016 | 67 | 70 | 0.955 | 0.869,0.985 | 0.0001 | 0% |
| Full strength Formocresol | Overall | 24 months | 1 | 2016 | 34 | 35 | 0.971 | 0.823,0.996 | 0.001 | N/A |
| Diluted Formocresol | Clinical | 6 months | 4 | 2008-2015 | 85 | 85 | 0.977 | 0.913,0.994 | 0.0001 | 0% |
| Diluted Formocresol | Clinical | 12 months | 4 | 2008-2015 | 81 | 85 | 0.935 | 0.799,0.981 | 0.0001 | 29.99% |
| Diluted Formocresol | Clinical | 24 months | 3 | 2008-2011 | 54 | 60 | 0.914 | 0.619,0.986 | 0.014 | 57.62% |
| Diluted Formocresol | Radiographic | 6 months | 4 | 2008-2015 | 83 | 85 | 0.948 | 0.860,0.982 | 0.0001 | 0% |
| Diluted Formocresol | Radiographic | 12 months | 4 | 2008-2015 | 78 | 85 | 0.884 | 0.768,0.946 | 0.0001 | 11.59% |
| Diluted Formocresol | Radiographic | 24 months | 3 | 2008-2011 | 51 | 60 | 0.827 | 0.670,0.918 | 0.0001 | 20.06% |
| Diluted Formocresol | Overall | 6 months | 3 | 2005-2015 | 75 | 75 | 0.981 | 0.910,0.996 | 0.001 | 0% |
| Diluted Formocresol | Overall | 12 months | 2 | 2011-2015 | 50 | 50 | 0.981 | 0.876,0.997 | 0.0001 | 0% |
| Diluted Formocresol | Overall | 24 months | 1 | 2011 | 25 | 25 | 0.981 | 0.756,0.999 | 0.006 | N/A |
| Calcium Hydroxide | Clinical | 6 months | 6 | 2000-2015 | 150 | 176 | 0.894 | 0.716,0.966 | 0.001 | 75.75% |
| Calcium Hydroxide | Clinical | 12 months | 6 | 2000-2015 | 136 | 176 | 0.853 | 0.593,0.959 | 0.013 | 86.24% |
| Calcium Hydroxide | Clinical | 24 months | 4 | 2005-2014 | 120 | 137 | 0.887 | 0.748,0.954 | 0.0001 | 59.57% |
| Calcium Hydroxide | Radiographic | 6 months | 4 | 2008-2015 | 60 | 94 | 0.638 | 0.453,0.789 | 0.14 | 63.31% |
| Calcium Hydroxide | Radiographic | 12 months | 6 | 2000-2015 | 100 | 176 | 0.579 | 0.341,0.786 | 0.52 | 87.04% |
| Calcium Hydroxide | Radiographic | 24 months | 4 | 2005-2014 | 94 | 137 | 0.711 | 0.446,0.882 | 0.11 | 85.20% |
| Calcium Hydroxide | Overall | 12 months | 10 | 2000-2012 | 162 | 257 | 0.632 | 0.515,0.736 | 0.03 | 66.03% |
| Calcium Hydroxide | Overall | 18 months | 6 | 2000-2014 | 106 | 163 | 0.647 | 0.480,0.784 | 0.08 | 72.35% |
| Calcium Hydroxide | Overall | 24 months | 5 | 2008-2012 | 64 | 148 | 0.43 | 0.318,0.549 | 0.251 | 46.61% |
| Ferric Sulphate | Clinical | 6 months | 6 | 2005-2017 | 150 | 155 | 0.942 | 0.849,0.979 | 0.0001 | 34.16% |
| Ferric Sulphate | Clinical | 12 months | 11 | 1991-2018 | 291 | 336 | 0.94 | 0.767,0.987 | 0.001 | 88.47% |
| Ferric Sulphate | Clinical | 24 months | 7 | 2005-2017 | 200 | 226 | 0.852 | 0.785,0.901 | 0.0001 | 0% |
| Ferric Sulphate | Radiographic | 6 months | 5 | 2011-2017 | 96 | 105 | 0.883 | 0.795,0.936 | 0.0001 | 0% |
| Ferric Sulphate | Radiographic | 12 months | 9 | 1991-2018 | 253 | 284 | 0.878 | 0.816,0.922 | 0.0001 | 28.52% |
| Ferric Sulphate | Radiographic | 24 months | 3 | 2005-2017 | 75 | 90 | 0.829 | 0.735,0.894 | 0.0001 | 0% |
| Ferric Sulphate | Overall | 6 months | 1 | 2011 | 25 | 25 | 0.981 | 0.756,0.998 | 0.006 | N/A |
| Ferric Sulphate | Overall | 12 months | 9 | 1991-2015 | 248 | 279 | 0.873 | 0.811,0.916 | 0.0001 | 23.44% |
| Ferric Sulphate | Overall | 18 months | 5 | 1997-2015 | 142 | 159 | 0.888 | 0.827,0.929 | 0.0001 | 0% |
| Ferric Sulphate | Overall | 24 months | 5 | 2005-2013 | 132 | 154 | 0.852 | 0.785,0.901 | 0.0001 | 0% |
| Sodium Hypochlorite | Overall | 12 months | 3 | 2013-2015 | 69 | 78 | 0.871 | 0.770,0.932 | 0.0001 | 0% |
| Sodium Hypochlorite | Overall | 18 months | 2 | 2013-2015 | 37 | 40 | 0.924 | 0.790,0.975 | 0.0001 | 0% |
| Biodentine | Clinical | 6 months | 7 | 2015-2017 | 210 | 212 | 0.978 | 0.945,0.991 | 0.0001 | 0% |
| Biodentine | Clinical | 12 months | 6 | 2016-2017 | 180 | 184 | 0.971 | 0.931,0.988 | 0.0001 | 0% |
| Biodentine | Clinical | 18 months | 3 | 2017 | 67 | 69 | 0.964 | 0.883,0.989 | 0.0001 | 0% |
| Biodentine | Radiographic | 6 months | 7 | 2015-2017 | 199 | 212 | 0.939 | 0.824,0.981 | 0.0001 | 67.61% |
| Biodentine | Radiographic | 12 months | 1 | 2016 | 43 | 45 | 0.956 | 0.839,0.989 | 0.0001 | N/A |
| Biodentine | Overall | 12 months | 2 | 2016 | 60 | 63 | 0.952 | 0.862,0.985 | 0.0001 | 0% |
| Portland Cement | Clinical | 6 months | 1 | 2016 | 33 | 35 | 0.943 | 0.798,0.986 | 0.0001 | N/A |
| Portland Cement | Clinical | 12 months | 1 | 2016 | 33 | 35 | 0.943 | 0.798,0.986 | 0.0001 | 0% |
| Portland Cement | Clinical | 24 months | 1 | 2016 | 33 | 35 | 0.943 | 0.798,0.986 | 0.0001 | 0% |
| Portland Cement | Radiographic | 6 months | 1 | 2016 | 35 | 35 | 0.981 | 0.813,0.999 | 0.0001 | 0% |
| Portland Cement | Radiographic | 12 months | 1 | 2016 | 35 | 35 | 0.981 | 0.813,0.999 | 0.0001 | 0% |
| Portland Cement | Radiographic | 24 months | 1 | 2016 | 31 | 35 | 0.886 | 0.732,0.964 | 0.001 | N/A |
| Diode Laser | Clinical | 6 months | 3 | 2014-2015 | 65 | 65 | 0.974 | 0.882,0.995 | 0.0001 | 0% |
| Diode Laser | Clinical | 12 months | 2 | 2014-2015 | 50 | 50 | 0.977 | 0.851,0.997 | 0.0001 | 0% |
| Diode Laser | Radiographic | 6 months | 3 | 2014-2015 | 57 | 65 | 0.862 | 0.751,0.928 | 0.0001 | 0% |
| Diode Laser | Radiographic | 12 months | 2 | 2014-2015 | 40 | 50 | 0.827 | 0.484,0.961 | 0.06 | 41.15% |
| Diode Laser | Overall | 12 months | 3 | 2014-2015 | 86 | 102 | 0.839 | 0.667,0.932 | 0.001 | 61.44% |
| Diode Laser | Overall | 18 months | 2 | 2014-2015 | 53 | 62 | 0.835 | 0.630,0.938 | 0.004 | 54.71% |
| Electro Surgery | Clinical | 6 months | 2 | 2014-2015 | 23 | 25 | 0.887 | 0.543,0.981 | 0.032 | 35.86% |
| Electro Surgery | Radiographic | 6 months | 2 | 2014-2015 | 20 | 25 | 0.8 | 0.6,0.914 | 0.006 | 0% |
| ABS | Clinical | 6 months | 2 | 2014-2017 | 50 | 50 | 0.979 | 0.866,0.997 | 0.0001 | 0% |
| ABS | Clinical | 12 months | 2 | 2014-2017 | 45 | 50 | 0.886 | 0.706,0.962 | 0.001 | 14.79% |
| ABS | Radiographic | 6 months | 2 | 2014-2017 | 48 | 50 | 0.941 | 0.622,0.994 | 0.017 | 54.43% |
| ABS | Radiographic | 12 months | 2 | 2014-2017 | 43 | 50 | 0.859 | 0.734,0.932 | 0.0001 | 0% |
| EMD | Clinical | 6 months | 2 | 2008-2016 | 46 | 50 | 0.919 | 0.804,0.969 | 0.0001 | 0% |

**Appendix 9: Risk of bias in the included systematic reviews as assessed by ROBIS tool.**

| SNo | Author | Year | RISK OF BIAS IN THE REVIEW | | | Final |
| --- | --- | --- | --- | --- | --- | --- |
|  |  |  | Describe whether conclusions were supported by the evidence: | | |  |
|  |  |  | A. Did the interpretation of findings address all of the concerns identified in Domains 1 to 4? | B. Was the relevance of identified studies to the review's research question appropriately considered? | C. Did the reviewers avoid emphasizing results on the basis of their statistical significance? |  |
|  |  |  | Y/PY/PN/N/NI | Y/PY/PN/N/NI | Y/PY/PN/N/NI |  |
| 1 | De Coster et al | 2012 | Probably No | Probably Yes | Probably Yes | High |
| 2 | Marghalani et al | 2014 | Probably No | Probably Yes | Probably Yes | High |
| 3 | Coll JA et al | 2017 | Yes | Probably Yes | Probably Yes | Low |
| 4 | Nematollahi et al | 2018 | Probably No | Probably Yes | Probably Yes | High |
| 5 | Nuvvula et al | 2018 | Probably Yes | Probably Yes | Probably Yes | Low |
| 6 | Smaïl-Faugeron et al | 2018 | Probably No | Probably Yes | Probably Yes | High |
| 7 | Stringhini Junior et al | 2019 | Probably No | Probably Yes | Probably Yes | High |
| 8 | Jayaraman et al | 2020 | Yes | Probably Yes | Probably Yes | Low |
